# First dynamics of bacterial community during development of *Acropora humilis* larvae in aquaculture

**DOI:** 10.1038/s41598-021-91379-w

**Published:** 2021-06-03

**Authors:** Chitrasak Kullapanich, Suppakarn Jandang, Matanee Palasuk, Voranop Viyakarn, Suchana Chavanich, Naraporn Somboonna

**Affiliations:** 1 Department of Microbiology, Faculty of Science, Chulalongkorn University, Phyathai Road, Pathumwan, Bangkok, 10330 Thailand; 2 Microbiome Research Unit for Probiotics in Food and Cosmetics, Chulalongkorn University, Pathumwan, Bangkok, 10330 Thailand; 3 Reef Biology Research Group, Department of Marine Science, Faculty of Science, Chulalongkorn University, Phyathai Road, Pathumwan, Bangkok, 10330 Thailand; 4 Center of Excellence for Marine Biotechnology, Chulalongkorn University, Pathumwan, Bangkok, 10330 Thailand

**Keywords:** Ecology, Microbiology

## Abstract

A symbiosis of bacterial community (sometimes called microbiota) play essential roles in developmental life cycle and health of coral, starting since a larva. For examples, coral bacterial holobionts function nitrogen fixation, carbon supply, sulfur cycling and antibiotic production. Yet, a study of the dynamic of bacteria associated coral larvae development is complicated owning to a vast diversity and culturable difficulty of bacteria; hence this type of study remains unexplored for *Acropora humilis* larvae in Thai sea. This study represented the first to utilize 16S rRNA gene sequencing to describe the timely bacterial compositions during successfully cultured and reared *A. humilis* larval transformation in aquaculture (gametes were collected from Sattahip Bay, Chonburi province, Thailand), from gamete spawning (0 h) and fertilization stage (1 h), to embryonic cleavage (8 h), round cell development (28, 39 and 41 h), and planula formation (48 h). The sequencing results as estimated by Good’s coverage at genus level covered 99.65 ± 0.24% of total bacteria. While core phyla of bacteria were observed (Proteobacteria, Actinobacteria, Firmicutes and Bacteroidetes), changes in bacterial population structures and differential predominant core bacterial orders were denoted for each larval developmental stage, from fertilization to embryonic cleavage and subsequently from the embryonic cleavage to round cell development (*P* = 0.007). For instances, *Pseudoalteromonas* and Oceanospirillales were found prevalent at 8 h, and Rhizobiales were at 48 h. The bacterial population structures from the round cell stage, particularly at 41 h, showed gradual drift towards those of the planula formation stage, suggesting microbial selection. Overall, this study provides preliminary insights into the dynamics of bacterial community and their potentially functional association (estimated from the bacterial compositions) during the developmental embryonic *A. humilis* in a cultivation system in Southeast Asia region.

## Introduction

In the past decades, stresses from climate warming and anthropogenic activities have dramatically affected worldwide coral reef health, including Thailand and Southeast Asia region^1,2^. Some coral genera, such as *Acroporidae*, *Faviidae*, *Pocilloporidae* and *Poritidae*, have significantly declined^3–5^. This decline affects ecologic and economic benefits of coral reefs, including damaged and declined marine life habitat and food sources, and disrupted coastal protection^6–8^. Additionally, many new bioactive compounds in human medicine have extracted from coral reef habitat^9–11^. Subsequently, scientists are interested in finding sustainable management for restoring and conserving coral reefs^12–14^.

One of the most recent focus to assist in coral reef restoration and conservation is through the coral-associated microbiome. Microorganisms, mainly bacteria, were reported to colonize various parts of coral tissues (mucus layer, gastrovascular cavity and skeleton) and function in coral growth, health and promoting the higher resistance to stresses that include climate warming^15,16^. For instances, many coral holobiont bacteria (e.g. *Vibrionales*, *Cyanobacteria*, *Alteromonas*, *Rhizobales, Oleibacter* and *Pseudoalteromonas*) recycle nutrients by nitrogen fixation, sulfur cycling and photosynthesis^17–19^, or synthesize bioactive compounds, such as tropodithietic acid (TDA), against pathogenic bacteria^20–22^. Subsequently, abundances of *Oceanospirillales* and *Rhizobiales* were reported to be associated with healthy corals of various species^23–27^. Yet, scientists found that these coral bacteria are dynamics through the coral developmental stages and external factors^28–30^. Damjanovic et al*.*^31^ reported the presence of bacteria since *Acropora tenuis* egg and sperm bundles, and the early coral life stages (0–96 h) were associated with a dynamic and diverse bacterial community; the initial bacteria on gametes could be from a vertical transfer of certain bacteria in the mucus layer surrounding the gametes and a release of bacteria by spawning adults into the water column (a horizontal transfer). Miller et al*.*^32^ found that bacterial communities of nursery-reared *Acropora cervicornis* were relatively consistent among spatial colonies of the same coral host genotype and varied by coral host genotypes. Still, the knowledge about the bacterial population structures during larval development of *Acropora humilis* in the Southeast Asia has not been deciphered.

The cultivation of corals using sexual reproduction technique is relatively new in the past decade with different levels of success depending on sites^33–37^, and we are one of the pioneer groups who have actually been successfully cultivating corals using gametes and have successfully been using our cultured baby corals to restore degraded reefs in Thailand. Here, *A. humilis* can be a model coral species since this species is a predominant species not only in Thailand but also in the tropical regions, particular in the Southeast Asia. However, not many studies had done on this coral species because the hardness of gamete and larval sample collection, and the limitation of the success in coral cultivation from gamete fertilization. At present, it is impossible to collect those wild fertilized coral gametes in the field, and thus, cultured corals in a hatchery represented an excellent alternative choice to study the biology of *A. humilis* species in larval stages. In addition, even the same genus or species of corals exist in different marine areas, dynamics of the bacterial populations are usually not the same due to different marine biogeography factors, which affect a quality of water, degree and period of sunlight, salinity, oxygen, pH, sedimentation, nutrients, pollutions, etc.^16,30,38^. In this study, we reported truly the dynamics of the bacterial population structures (and their functional potentials) during the developing *A. humilis* larvae in aquaculture using metagenomic-derived 16S rRNA gene sequencing. This important knowledge will increase the understanding of the relationship between the bacterial communities and larval development for further apply in coral sexual reproduction cultivation technique.

## Material and methods

### Sample collections and coral larvae aquaculture

In February 2018, the gametes of five spawning coral colonies of *A. humilis* were collected from Sattahip Bay, Chonburi Province, Thailand. *A. humilis* is a hermaphroditic species, which spawns once a year. At Sattahip Bay, the spawning times are usually between 7:30–9:00 pm. For fertilization, collecting gametes from five different coral colonies have been proved to be sufficient for gamete fertilization succession rate at 98–99%. Fertilization rate is determined by random collecting of approximately 100 individuals of eggs after 24 h of encountering with sperms, and counting on fertilized eggs using a dissecting microscope. The average fertilization rates are based on at least 3 replicates. To collect the gametes, the scuba diving technique was used; the divers covered a plankton net in each coral colony before the coral spawning approximately 30 min. Then, after collection, the gametes were immediately transferred to a hatchery facility, mixed, and artificially fertilized. Once gametes were fertilized, they were transferred to 30 × 60 × 30 cm^3^ tanks filled with 54 L of 0.4 µm filtered seawater at ambient temperature (28 °C), ambient salinity (32 psu), and day-night cycle via natural light intensity of 21 µmol m^-2^ s^-1^ for 12 h a day. The density of larvae was < 2000 larvae/L of seawater. The filtered seawater was exchange daily to prevent contaminated growth of other organisms. The fertilization and coral raising techniques were followed Kuanui et al*.*^39,40^. Those gametes were fertilized and developed in filtered seawater in the enclosed system. Thus, the variation and the influence of microorganisms from the sea environment should be limited. To exchange the seawater, one-third of the seawater volume were replaced each time to ensure that the fertilized gametes were not disturbed. At specific time points including gamete spawning (0 h) and fertilization stage (1 h), to embryonic cleavage (8 h), round cell development (28 h, 39 h and 41 h), and planula formation (48 h), three replicates of fertilized gamete samples were collected. At 0 h meant the stage of unfertilized eggs prior to be mixed with other colonies for fertilization.

### Metagenomic extraction

Protocols for DNA extraction and 16S rRNA gene sequencing were followed https://earthmicrobiome.org/protocols-and-standards/dna-extraction-protocol/^41,42^. Metagenomic DNA were extracted from 1 g of each ground (consisting of endoderm and ectoderm) larvae sample using Power Soil DNA Isolation Kit (Qiagen Inc., Hilden, Germany). Protocols were as described in the manufacturer’s instructions. The extracted metagenomes were checked for DNA quality and concentration by agarose gel electrophoresis (1% agarose gel) and nanodrop spectrophotometry (A_260_/A_280_ and A_260_)^42^. Noted that some DNA samples were found degraded and could not successfully amplified for library construction at the next step. For preservation, the extracted DNA were stored at -20 °C.

### 16S rRNA gene library construction and next generation sequencing

The extracted DNA were used as templates for a preparation of prokaryotic 16S rRNA gene V3–V4 libraries. Amplification was performed using a universal forward primer 515F (5’-GTGCCAGCMGCCGCGGTAA-3’) and a universal reverse primer 806R (5’-GGACTACHVGGGTWTCTAAT-3’), with appended Illumina adapter and Golay barcode sequences^42,43^. Each biological replicate (n = 3 per sampling time point) was amplified in triplicate PCR reactions to prevent a PCR bias. Each PCR (25 µL) comprised 1 × EmeraldAmp GT PCR Master Mix (TaKaRa, Shiga, Japan), 0.3 µM of each primer, and 50 ng metagenomic DNA template. The amplification parameters included an initial heat activation at 94 °C for 3 min, and 25 cycles of denaturation at 94 °C for 45 s, annealing at 50 °C for 60 s, and extension at 72 °C for 1.5 min, followed by a final extension at 72 °C for 10 min. The PCR products of expected 381 bp in size were purified using Gel Extraction Kit (Bio-Helix Co. Ltd., Keelung, Taiwan). Triplicate PCR products were combined equally and quantified using Qubit 3.0 Fluorometer and Qubit dsDNA HS Assay Kit (Thermo Fisher Scientific Inc., Massachesetts, USA). 150 ng of each sample was sequenced along sequencing primer and index sequence^43^, on a Miseq platform with 2 × 150 bp paired-end reads (Illumina, California, United States) housed at Omics Science and Bioinformatics Center, Faculty of Science, Chulalongkorn University (Bangkok, Thailand).

### Bioinformatic and statistical analyses

Raw sequences (reads) were quality screened following Mothur’s standard operating procedures (SOP) for MiSeq, which included removal of primer sequences, short read length (< 100 bp), read with ambiguous bases, and chimera sequences^44,45^. The quality reads were then aligned against GreenGenes 13.8 and SILVA 1.32 databases to remove mitochondria, chloroplast, unknown and eukaryote sequences, and clustered into operational taxonomic units (OTUs) at phylum, class, order, family and genus levels. Nucleic acid sequences in this study were deposited to an open access Sequence Read Archive database of NCBI, accession number SRP275543.

Mothur was used to compute Good’s coverage (an estimate of sequencing depth to the true diversity in a sample) and alpha diversity (Chao richness, Shannon diversity and inverse Simpson indices)^44,45^. To obtain an equal sequencing depth across samples, the lowest quality read number (7,184 reads) per sample was used for data rarefication and continuing data analyses. Noted that samples with unsuccessful metagenomic extraction or insufficient sequencing depth (< 99% sequencing coverage) were removed from further analyses to prevent bias in representing the true community composition^46,47^. Mothur was also used to compute beta diversity (thetayc dissimilarity index), a phylogenetic tree (neighbour joining tree), and functional potentials estimated from bacterial community profiles via PICRUSt (Phylogenetic Investigation of Communities by Reconstruction of Unobserved States). The PICRUSt categorizes functions into KEGG (Kyoto Encyclopedia of genes and genomes) pathways^48^. For statistical analysis, Wilcoxon signed rank test and analysis of molecular variance (AMOVA) were used to test for significant difference between groups (*P* < 0.05). Data visualization and statistical analyses were conducted using RStudio 1.3.959 (http://www.rstudio.com/)^49^.

## Results

### 16S rRNA gene sequencing

**Figure 1:**
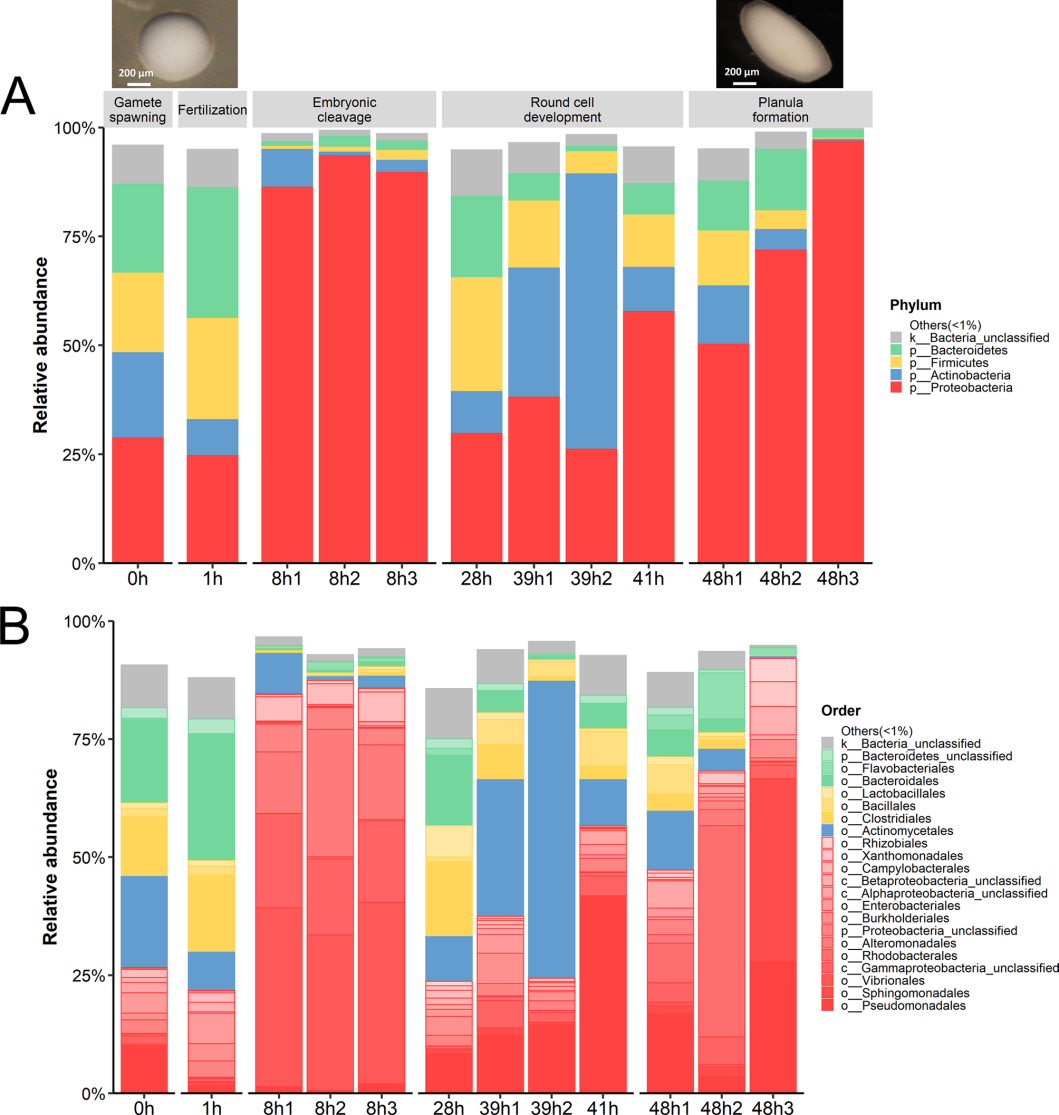
Relative abundance of bacterial OTU compositions during *A. humilis* larval development in aquaculture at (**A**) phylum and (**B**) order levels. Bacterial phyla and orders with < 1% abundance were represented in “Other (< 1%)”. In (**A**), pictures corresponding to 1 h and 48 h *A. humilis* larvae were included.

The libraries of our 16S rRNA gene sequences correspoding to samples named 0h, 1h, 8h1, 8h2, 8h3, 28h, 39h1, 39h2, 41h, 48h1, 48h2 and 48h3 passed quality screening and OTU classifications. An average number of quality reads were 16,926 reads per sample. At genus level OTUs, these numbers of quality reads passed > 99% Good’s coverage (Supplemental Table 1). Overall, four phyla were observed (Proteobacteria averagely 57.90%, Actinobacteria 14.23%, Firmicutes 10.11% and Bacteroidetes 8.08%), and each stage of *A. humilis* larval development carried different bacterial diversity. For alpha diversity, larvae samples at 8 h showed noticably low bacterial diversity compared to the other time points (*P* = 0.009). Figure 1A showed the consistently least alpha diversity among 8h1, 8h2 and 8h3 (Supplemental Fig. 1A showed an average bacterial composition of the 8 h samples). The 8 h bacteria were dominated by phylum Proteobacteria (89.95%), specifically in order Vibrionales (36.38%) followed by Alteromonadales (18.61%) and an unclassified order in class Gammaproteobacteria (17.76%), in orderly (Supplemental Fig. 1A). Interestingly, these species of genera *Pseudoalteromonas* and *Oleibacter* belonged an order Oceanospirillales (Supplemental Fig. 2A). Raw numeric percentages of bacterial genus compositions of all samples were available in Supplemental Table 2.

**Figure 2:**
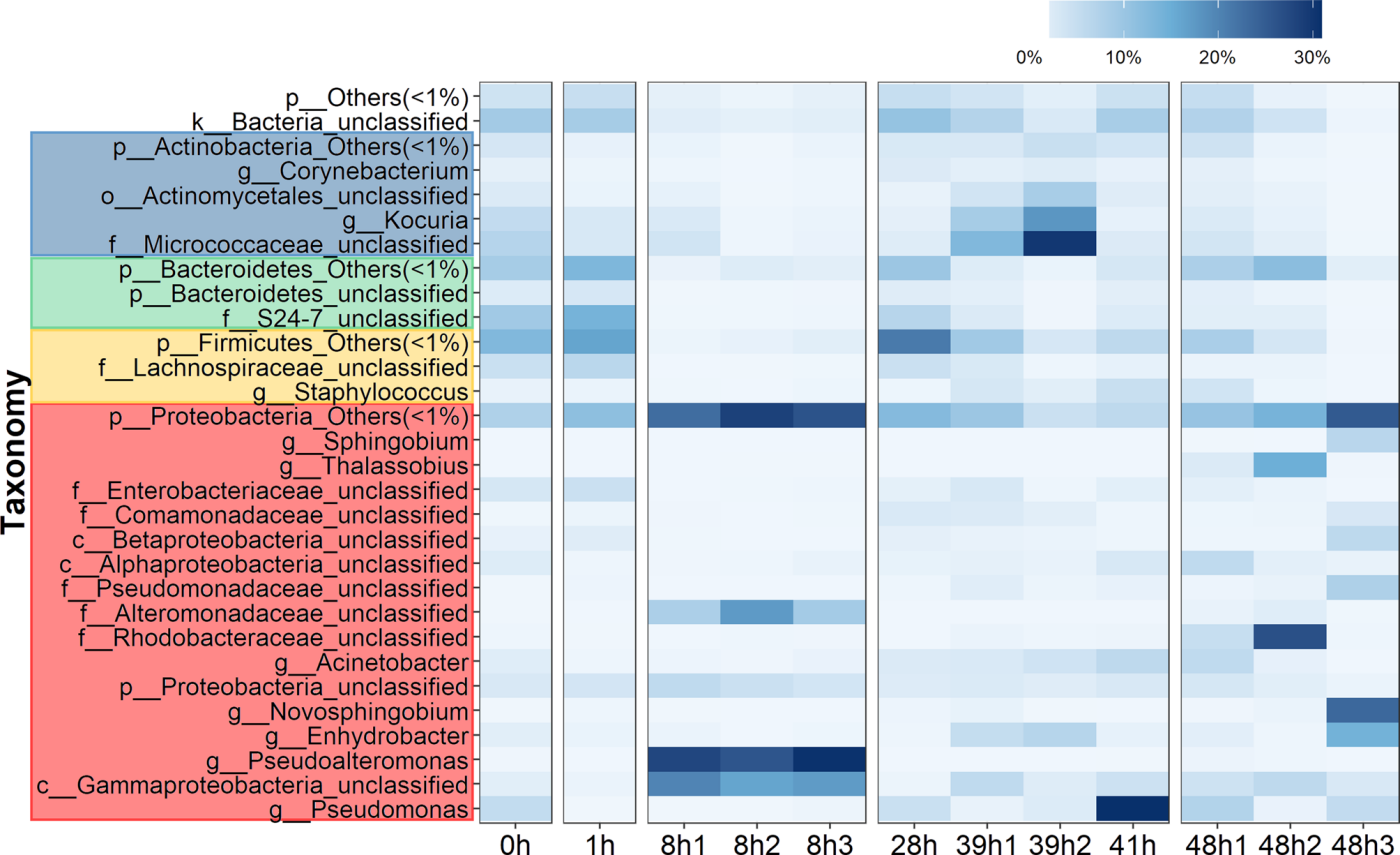
Relative abundance of bacterial OTU compositions at genus level. For OTU classification where genus could not be identified, the deepest classification was given (abbreviated g_ for genus, f for family, c for class and o for order, respectively). Genus names were color highlighted based on phylum, and in each phylum bacterial genera of < 1% abundance were represented in “p_name_Others (< 1%)”.

### Comparative bacterial community profiles during larval development

Figure 1A displayed interesting pattern in the bacterial phyla including the relatively even distributions of four core phyla during 0 and 1 h larvae, the drastic increase of *Proteobacteria* but almost diminished other phyla in samples 8h1 to 8h3, and the reversion of bacterial population structure in 28 h to be resembling that of 1 h (e.g. increase of Firmicutes and Bacteroidetes) before continuiation of bacterial dynamics such as a transient increase of Actinobacteria at 39h1 and 39h2, and an increasing of Proteobacteria at 48h1 to 48h3. Noted a test of statistical significance between each pairwise time points was impossible because of insufficient sample size. Beta diversity analyses (dissimilarity of bacterial community structures among samples) indicated relatively closer bacterial population structures between stages of gamete spawning and fertilization, and round cell development and planula (*P* = 0.055). For the latter, increasing relative abundance of genera *Magnetospirillum* and unclassified Rhizobiales that belong order Rhizobiales were denoted (Supplemental Fig. 2B, averagely 1.98% for 48 h vs. 0.33% for the rests).

**Figure 3:**
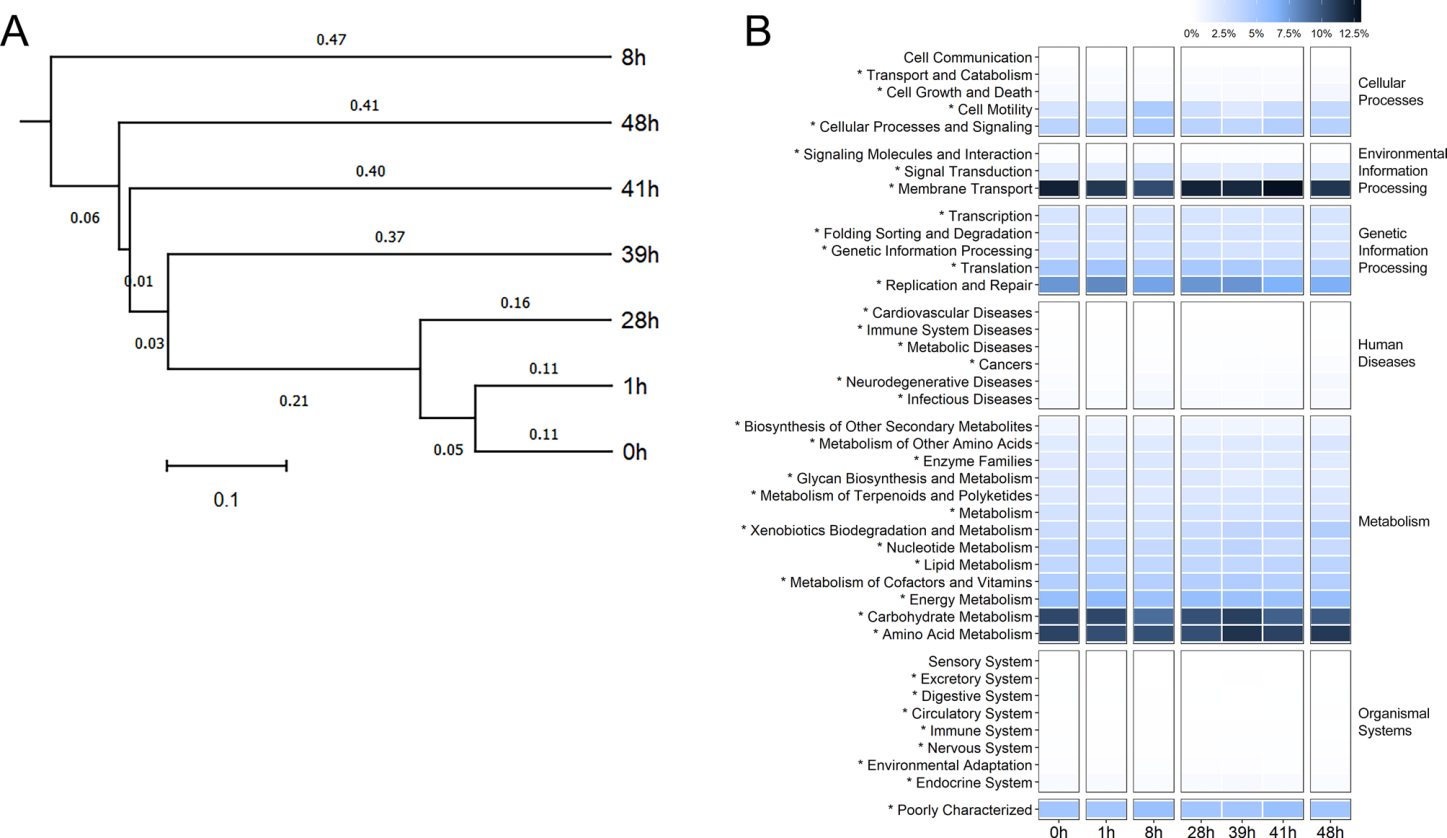
Analyses of bacterial community profiles at genus level OTUs into (**A**) phylogenetic tree and (**B**) microbial functional potentials. In (**B**), * denotes a functional category that had a statistical difference in relative abundance across samples.

Further diversity analysis at order level in Fig. 1B revealed the orders that correlated with each coral developmental stages. This included the drastic change at 8 h (embryonic cleavage), mainly comprising Vibrionales (36.38%), followed by Alteromonadales (18.61%) and an unclassified order in class Gammaproteobacteria (17.76%). During round cell development, order Actinomycetales, of family Micrococcaceae and genus *Kocuria*, were prevalent (Figs. 1B and 2). Following at 41 h, these bacteria members were replaced by members of Proteobacteria, such as *Novosphingobium* and *Enhydrobacter* (Fig. 2 and Supplemental Fig. 1B). Furthermore, analysing at order and genus levels also revealed differences in relative abundances of bacterial members in phylum Proteobacteria in 8 h vs. 48 h. For instances, genera *Novosphingobium*, *Thalassobius*, *Enhydrobacter* and an unclassified in family Rhodobacteraceae were significantly higher in 48 h (Fig. 2). This difference along the presence of more diversified Bacteroidetes and Firmicutes, in 48 h larval bacterial population structures made the 48h1 to 48h3 cluster closer to the rest of the bacterial population structures than the 8h1 to 8h3 bacterial population structures (Fig. 3A).

Moreover, abundances of *Oceanospirillales* and *Rhizobiales* were compared. The greater relative abundance of Oceanospirillales in 8 h and Rhizobiales in 48 h, respectively, supported healthy status of our aquacultured corals and suggested their functions be necessary for the coral activities during these points (Supplemental Fig. 2)^23–27^. Note a somewhat diversity among the 28–41 h bacterial communities in part supported the detail differences in larval morphological development within this time period, such as round vs. tear drop stages.

### Functional potentials of bacterial communities

The bacterial communities of our cultured *A. humilis* larval development demonstrated core microbial functional potentials in membrane transport, carbohydrate and amino acid metabolisms, replication and repair, energy metabolism, cellular processes and signaling, cell motility, and lipid and nucleotide metabolisms, in orderly. In detail differences, functions involved membrane transport, genetic replication and repair, and carbohydrate and amino acid metabolisms were predominated in coral gamete spawning and fertilization; cell motility, cellular processes, and cellular signaling in embryonic cleavage; membrane transport, genetic replication and repair, and carbohydrate and amino acid metabolisms in round cell development; and xenobiotic biodegradation and metabolism in planula (Fig. 3B). Examples of correlation between bacterial compositions and the metabolic potentials are from 39 to 48 h larvae that the increased relative abundance of Rhodobacteraceae and *Novosphingobium*, which perform role as nitrogen recycler, and nitrogen is essential for coral growth from a transformation of planula into fully developed polyps^50,51^; thereby relatively high amino acid metabolism were highlighted.

## Discussion

Presence of bacteria had been reported in brooded coral larvae^52^. But, how a marine biogeography factor (i.e. Thai sea) plays role on coral gamete development has never been described and yet neither for the prevalent *A. humilis* species in the Thai sea, and this study is the first to identify the bacterial compositions associated with the early coral development stages from gamete fertilization to the formation of planula in aquacultured *A. humilis*. Little information were available on bacteria associated with corals particular at the young stages and larval stages, and none for the *A. humilis* gamete, due to the limitation and availability of sample collection, sexual reproduction and culture techniques, and advanced sequencing technique. Scientists reported that periods of day and night lights, tides and temperature have effects on *A. humilis* spawning; hence successful culturing of Thai sea *A. humilis* since gametes likely involves particular microbial association, and how the developing corals select certain bacteria from the surroundings (Thai sea reef ecosystem, in this case) remain unknown^53–55^.

Our findings of four core phyla through cultured *A. humilis* larval development were similar to those reported in *Acropora digitifera* at 48 h and 4 day larval stages, and that Bernasconi et al*.*^30^ also reported the relatively higher abundance of *Bacteroidetes* than *Firmicutes*. Nonetheless, this latter report was not found consistent in our results. This finding highlights the differences by different coral species, different hour stages and that the surrounding Thai sea could also provide a differently bacterial population involvement^16,56,57^, so the bacterial associations found in our study were not completely same to the previous studies. Our findings also highlight the essential to study the specific coral species at specific marine site to help understand the bacterial involvement and bacterial manipulation in enhancing *A. humilis* larval growth in the Thai sea. In 0 and 1 h bacteria, relative abundances of unclassified Alphaproteobacteria, Gammaproteobacteria and order Actinomycetales were consistent with previous findings^29^.

The relatively lowest alpha diversity at 8 h might support the dynamic point of coral bacteria from fertilization to cell rounding stages following Zhou et al*.*^29^ who reported an increasing bacterial diversity during larvae to juvenile. In addition, the 8 h bacterial population structures were uniquely distinct from the other samples (Figs. 1 and 2), providing bacteria to support functions in cell motility and cellular processes and signaling (Fig. 3B).

During round cell development, prevalent species in family Micrococcaceae and genus *Kocuria* that we observed had been described^58^ and might support the observed larval bacterial biofilm and function in quarum sensing to induce specific bacterial and/or substrate attachment essential for larval development. In many marine invertebrates whose the development of larvae involves a settlement on specific substrates to initiate metamorphosis to juvenile form, such as *Hydractinia symbiolongicarpus* (a cnidaria hydroid that grows on a gastropod shell), the bacterial isolates *Microbacterium* and *Kocuria* could synthesize and secrete quorum sensing signaling molecule, for example in their bacterial biofilms on coral larvae. This signal molecule could participate in the coral substrate selection by inducing larval chemotaxis and substrate attachment^58^. Further, species in Micrococcaceae, such as *Micrococcus* MCCB104, had been described to produce antimicrobial compounds against pathogenic bacteria (e.g. *Vibrio* spp.) and prevent disease in prawns and other marine organisms^59^.

Recently, there have been evidences that during the coral oocytes’ stages, some symbiotic microorganisms, such as algae (photosynthetic eukaryotic microbes), were found inside, and could function as an additional nutrition and energy source for metabolism during the oocyte development^60^. Subsequently, there were studies that investigated original sources of symbiotic algae and the other microorganisms, and how they could enter to the oocyte membranes^61^. Moreover, on an ectoderm, symbiotic bacteria could help prevent gamete and embryo from pathogenic microbe inhabitation and infection^62,63^.

In conclusion, our bacterial community and metabolic potential analyses among 0–1 h, 8 h, 28–41 h and 48 h suggested coral-bacteria association to correlate with nutrient metabolism and genetic information processing in gamete spawning and fertilization, cellular processes and microbial selection in embryonic cleavage, environmental information processing in round cell development and a greater nitrogen supply in planula formation. Nonetheless, bacterial transcriptome study should be future performed to confirm our speculation. *Pseudoalteromonas* in 8 h and *Pseudomonas* in 41–48 h were reported to inhibit coral pathogens^64,65^, and Rhodobacteraceae involve reproduction and ontogeny of various coral species, i.e. *Acropora tenuis*, *Acropora digitifera*, *Pocillopora damicornis* and *Pocillopora acuta*^30,38,53,66,67^. In addition, orders Oceanospirillales and Rhizobiales were essential coral holobionts worldwide. Subsequently, as Oceanospirillales function in degradation of dimethylsulfoniopropionate (DMSP) into usable carbon and sulfur forms and also produces antimicrobial compounds, so their predominance at 8 h might support coral growth and maintianance of healthy coral status by preventing pathogenic bacteria^21,22^. During the latter larval development, in particular planula, Rhizobiales function in nitrogen fixation, so their predominance might support amino acid (protein) synthesis and hence the coral growth^28^. Neave et al*.*^68^ also reported functional specificity and diversification in developing *A. humilis* associated Oceanospirilllales genomes to be more enriched for functions in carbohydrate and protein cyclings. Note the large variation between 39h1 and 39h2 independent replicates perhaps suggested an effect of transitioning bacterial habitation in shaping the holobionts^69^, or an effect from natural variation among corals. Similarly, 48h1 and 48h2 (but 48h3) replicates showed a more community similarity to the 41 h, supportive of ongoing transitioning stage; the shortcoming of our preliminary study is a limited number of sequencing replicates to allow consensus evaluation of relative abundances of bacteria during this coral transitioning time points. Our findings of bacterial association and their dynamics strengthen the assertion of bacterial involvement in coral development and the perspective in using bacterial manipulation in enhancing coral growth. The knowledge from this study can be a baseline data and helped improve an intervention in cultivation of corals through sexual reproduction to enhance the coral growth and juvenile settlement in a hatchery for mass culture to support sustainable coral management.

## Supplementary Information

Supplementary Information.
