# Supplementary material for: First dynamics of bacterial community during development of Acropora humilis larvae in aquaculture
Source: Sci Rep. 2021 Jun 3;11:11762. doi: 10.1038/s41598-021-91379-w (PMC8175334; doi:10.1038/s41598-021-91379-w)
Supplement: Supplementary file 1 — Supplementary Information. [file 41598_2021_91379_MOESM1_ESM.docx]

**First dynamics of bacterial community during development of *Acropora humilis* larvae in aquaculture**

Chitrasak Kullapanich ^1,2^, Suppakarn Jandang ^3^, Matanee Palasuk ^1,2^, Voranop Viyakarn ^3^, Suchana Chavanich ^3,4*^, Naraporn Somboonna ^1,2*^

**Supplementary Figures and Tables**

**Supplemental Figure 1** Relative abundance of individual and (if having independent sample replicates) average bacterial OTU compositions during *A. humilis* larval development in aquaculture at (**A**) order and (**B**) genus levels.

In (A), bacterial orders and genera with < 1% abundance were represented in “Others (< 1%)”. For OTU classification where genus could not be identified, the deepest classification was given (abbreviated g_ for genus, f for family, c for class and o for order, respectively). Genera names were color highlighted based on phylum.


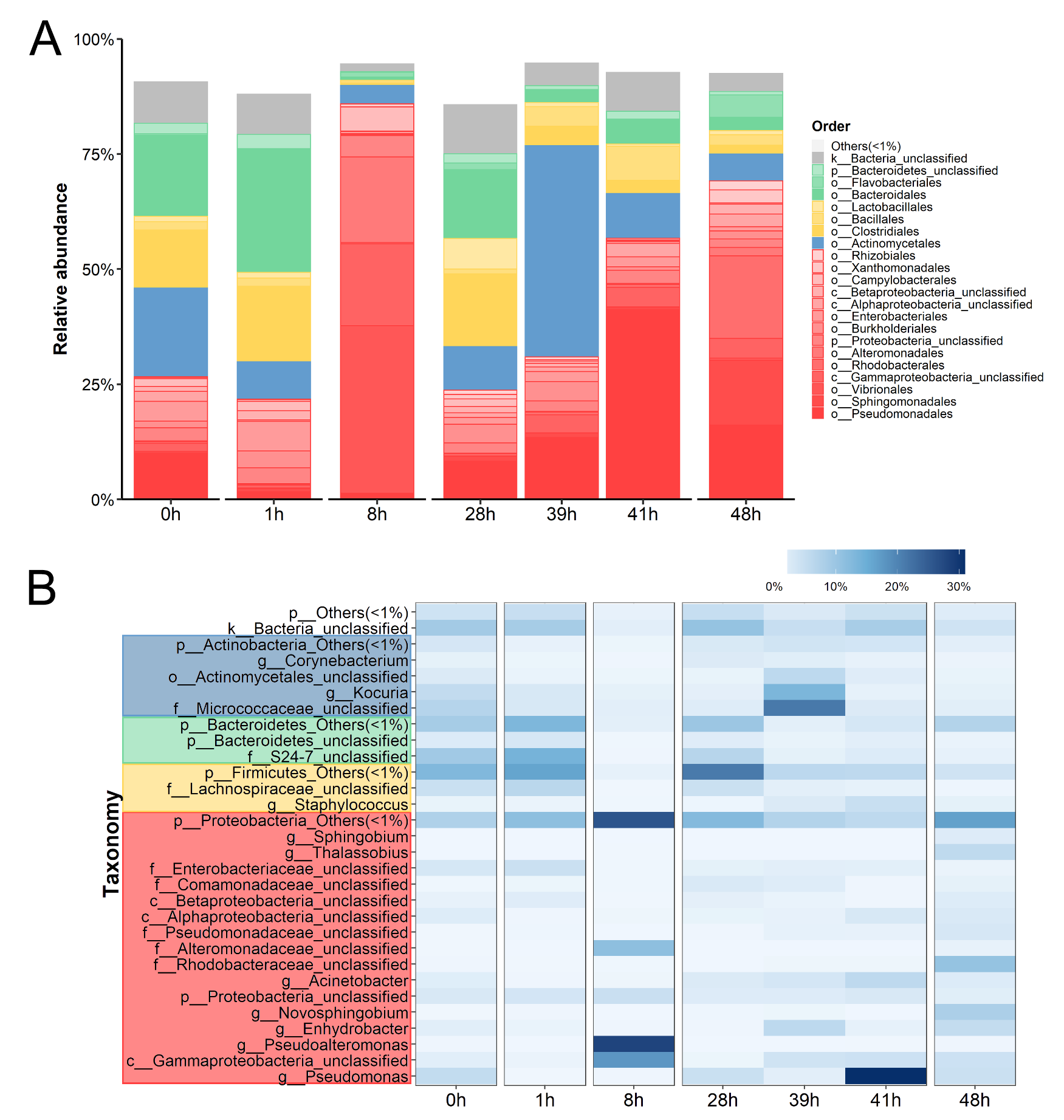


**Supplemental Figure 2** Relative abundance of bacterial orders associated with healthy corals, (**A**) *Oceanospirillales* and (**B**) *Rhizobiales*.


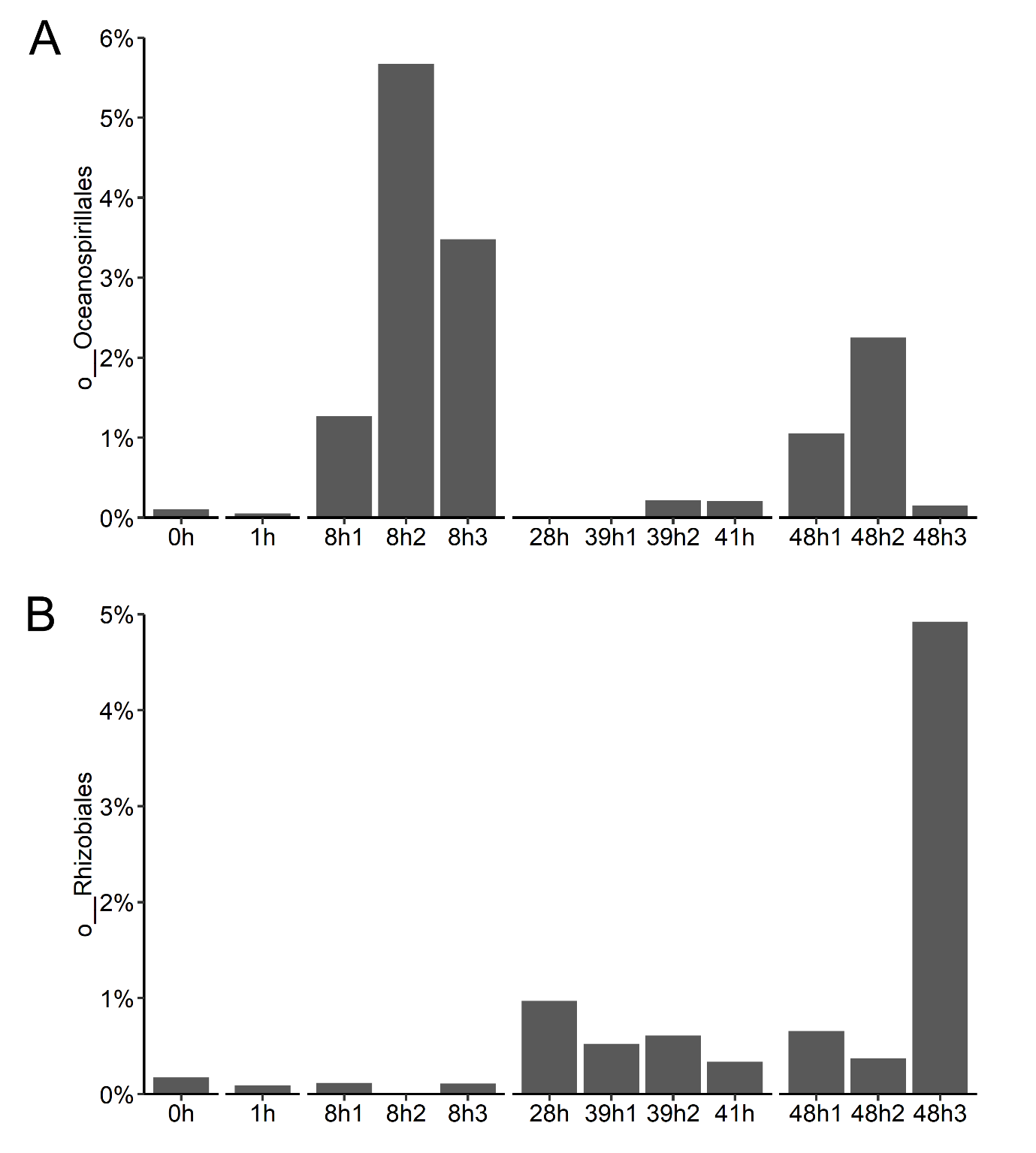


**Supplemental Table 1** Numbers of raw and quality reads, Good’s coverage, and alpha diversity indices (at genus level).

| Samples | Raw reads | Quality reads | Good's Coverage | OTUs | Chao | Shannon | Inverse Simpson |
| --- | --- | --- | --- | --- | --- | --- | --- |
| 0h | 16,049 | 10,362 | 99.48% | 242 | 282.89 | 3.9049 | 26.3731 |
| 1h | 14,503 | 9,801 | 99.40% | 215 | 296.48 | 3.7896 | 22.2076 |
| 8h1 | 54,473 | 18,645 | 99.71% | 187 | 248.88 | 2.6712 | 7.2608 |
| 8h2 | 30,618 | 8,467 | 99.48% | 140 | 177.84 | 2.6479 | 7.4458 |
| 8h3 | 25,227 | 7,184 | 99.18% | 184 | 241.03 | 2.8257 | 7.0974 |
| 28h | 21,902 | 18,968 | 99.89% | 201 | 216.00 | 4.0780 | 31.5732 |
| 39h1 | 28,796 | 22,889 | 99.92% | 146 | 163.00 | 3.6702 | 22.0673 |
| 39h2 | 31,311 | 28,212 | 99.87% | 231 | 275.40 | 2.8391 | 7.2085 |
| 41h | 42,647 | 21,004 | 99.86% | 226 | 244.91 | 3.2387 | 8.5905 |
| 48h1 | 52,776 | 14,920 | 99.62% | 286 | 352.96 | 4.1586 | 32.9412 |
| 48h2 | 70,307 | 12,131 | 99.51% | 252 | 307.19 | 3.2800 | 9.3859 |
| 48h3 | 32,885 | 30,530 | 99.92% | 138 | 150.50 | 2.8465 | 9.8875 |

**Supplemental Table 2** Raw numeric percentages (0.000-1.000) of bacterial genus compositions of 0h, 1h, 8h1, 8h2, 8h3, 28h, 39h1, 39h2, 41h, 48h1, 48h2 and 48h3. Noted that bacterial genera that could be identified are indicated with “g_” as prefix, while those that could not be identified genera are indicated with prefixes “k_”, “c_”, “o_” or “f_” to represent their deepest identifiable taxonomic levels (kingdom, class, order, or family).

| **Genus** | **0h** | **1h** | **8h1** | **8h2** | **8h3** | **28h** | **39h1** | **39h2** | **41h** | **48h1** | **48h2** | **48h3** |
| --- | --- | --- | --- | --- | --- | --- | --- | --- | --- | --- | --- | --- |
| **g__Pseudoalteromonas** | 0.212 | 0.031 | 27.879 | 25.877 | 30.610 | 0.026 | 0.022 | 0.053 | 0.067 | 0.597 | 0.289 | 0.010 |
| **c__Gammaproteobacteria** | 1.747 | 0.633 | 19.946 | 16.098 | 17.233 | 0.585 | 5.776 | 2.127 | 4.242 | 4.162 | 5.919 | 2.856 |
| **f__Micrococcaceae** | 7.035 | 2.928 | 3.781 | 0.319 | 0.821 | 2.077 | 12.801 | 29.899 | 2.409 | 3.579 | 1.673 | 0.174 |
| **k__Bacteria** | 9.014 | 8.775 | 1.829 | 1.394 | 1.726 | 10.623 | 7.134 | 2.705 | 8.446 | 7.400 | 3.924 | 0.403 |
| **g__Pseudomonas** | 5.424 | 0.092 | 0.081 | 0.095 | 0.473 | 4.592 | 0.913 | 2.371 | 30.918 | 7.312 | 0.808 | 5.500 |
| **g__Kocuria** | 5.501 | 2.949 | 2.682 | 0.295 | 0.710 | 1.429 | 8.694 | 17.840 | 1.147 | 2.580 | 1.171 | 0.075 |
| **f__S24-7** | 9.313 | 13.917 | 0.166 | 0.142 | 0.265 | 6.759 | 2.517 | 0.195 | 2.328 | 1.729 | 1.698 | 0.020 |
| **f__Alteromonadaceae** | 0.087 | 0.041 | 8.029 | 17.326 | 8.964 | 0.016 | 0.004 | 0.018 | 0.024 | 0.905 | 1.978 | 0.138 |
| **p__Proteobacteria** | 2.857 | 3.469 | 5.873 | 4.571 | 3.522 | 2.262 | 2.556 | 2.013 | 2.866 | 3.217 | 1.797 | 0.668 |
| **f__Rhodobacteraceae** | 0.328 | 0.061 | 0.048 | 0.260 | 0.223 | 0.016 | 0.620 | 0.323 | 0.457 | 4.960 | 26.519 | 0.301 |
| **g__Enhydrobacter** | 1.747 | 0.786 | 0.633 | 0.047 | 0.376 | 0.285 | 5.243 | 6.820 | 1.152 | 1.602 | 0.305 | 14.216 |
| **g__Novosphingobium** | 0.068 | 0.286 | 0.064 | 0.000 | 0.014 | 0.427 | 0.979 | 0.106 | 0.038 | 0.235 | 0.610 | 23.423 |
| **g__Acinetobacter** | 1.959 | 0.265 | 0.408 | 0.189 | 0.793 | 2.525 | 2.691 | 3.903 | 5.899 | 5.818 | 1.195 | 0.023 |
| **o__Actinomycetales** | 2.451 | 0.735 | 1.180 | 0.071 | 0.278 | 0.907 | 3.657 | 8.443 | 2.004 | 1.649 | 0.882 | 0.069 |
| **f__Lachnospiraceae** | 4.574 | 6.408 | 0.038 | 0.095 | 0.167 | 4.518 | 2.809 | 0.266 | 1.162 | 0.610 | 0.627 | 0.007 |
| **c__Alphaproteobacteria** | 2.200 | 0.296 | 0.198 | 0.295 | 0.877 | 0.986 | 1.236 | 0.851 | 2.928 | 5.778 | 1.492 | 0.960 |
| **g__Thalassobius** | 0.000 | 0.000 | 0.027 | 0.106 | 0.084 | 0.011 | 0.000 | 0.000 | 0.000 | 2.500 | 14.764 | 0.229 |
| **f__Enterobacteriaceae** | 3.185 | 4.510 | 0.054 | 0.165 | 0.251 | 1.344 | 3.006 | 0.319 | 1.595 | 1.528 | 0.618 | 0.016 |
| **g__Staphylococcus** | 0.955 | 0.673 | 0.134 | 0.024 | 0.362 | 0.369 | 3.220 | 1.794 | 4.809 | 3.767 | 0.363 | 0.030 |
| **o__Vibrionales** | 0.058 | 0.051 | 6.436 | 4.547 | 4.454 | 0.000 | 0.000 | 0.000 | 0.014 | 0.114 | 0.132 | 0.088 |
| **g__Alteromonas** | 0.039 | 0.000 | 2.607 | 5.941 | 4.051 | 0.000 | 0.000 | 0.000 | 0.010 | 0.275 | 0.618 | 0.036 |
| **f__Pseudomonadaceae** | 0.106 | 0.439 | 0.016 | 0.024 | 0.042 | 0.343 | 1.818 | 0.535 | 1.062 | 0.389 | 0.948 | 7.842 |
| **p__Bacteroidetes** | 2.297 | 3.081 | 0.097 | 0.130 | 0.195 | 2.019 | 1.507 | 0.234 | 1.662 | 1.609 | 0.627 | 0.098 |
| **c__Betaproteobacteria** | 1.042 | 2.051 | 0.145 | 0.012 | 0.028 | 1.413 | 0.861 | 0.695 | 0.352 | 0.268 | 0.280 | 6.007 |
| **o__Bacteroidales** | 2.750 | 3.653 | 0.043 | 0.095 | 0.111 | 1.961 | 0.524 | 0.142 | 1.662 | 0.905 | 0.503 | 0.026 |
| **f__Comamonadaceae** | 0.174 | 0.490 | 0.198 | 0.012 | 0.139 | 2.726 | 2.517 | 1.631 | 0.338 | 0.369 | 0.495 | 3.017 |
| **f__Ruminococcaceae** | 1.872 | 3.081 | 0.054 | 0.000 | 0.125 | 3.727 | 0.922 | 0.184 | 0.781 | 0.865 | 0.437 | 0.016 |
| **g__Arcobacter** | 0.405 | 0.000 | 3.535 | 3.165 | 4.552 | 0.000 | 0.009 | 0.035 | 0.000 | 0.047 | 0.017 | 0.000 |
| **g__Corynebacterium** | 1.274 | 0.561 | 0.274 | 0.024 | 0.278 | 2.409 | 1.512 | 2.166 | 1.043 | 1.428 | 0.338 | 0.020 |
| **g__Bacteroides** | 2.249 | 3.979 | 0.048 | 0.213 | 0.237 | 0.902 | 0.835 | 0.103 | 0.262 | 0.744 | 0.330 | 0.010 |
| **g__Rothia** | 1.255 | 0.469 | 0.290 | 0.024 | 0.153 | 0.991 | 1.219 | 1.861 | 1.228 | 1.079 | 0.107 | 0.016 |
| **g__Nonlabens** | 0.000 | 0.000 | 0.000 | 0.000 | 0.014 | 0.000 | 0.000 | 0.000 | 0.000 | 1.146 | 7.436 | 0.039 |
| **o__Bacillales** | 0.434 | 0.520 | 0.064 | 0.106 | 0.139 | 0.475 | 1.708 | 0.922 | 2.223 | 1.723 | 0.239 | 0.013 |
| **g__Vibrio** | 0.010 | 0.000 | 3.148 | 2.173 | 3.048 | 0.000 | 0.000 | 0.000 | 0.029 | 0.007 | 0.041 | 0.043 |
| **f__Moraxellaceae** | 0.521 | 0.061 | 0.107 | 0.118 | 0.167 | 0.390 | 1.638 | 0.890 | 1.881 | 1.622 | 0.371 | 0.252 |
| **g__Streptococcus** | 0.598 | 0.561 | 0.022 | 0.012 | 0.278 | 4.745 | 1.040 | 0.135 | 0.048 | 0.208 | 0.165 | 0.079 |
| **p__Firmicutes** | 1.506 | 2.010 | 0.043 | 0.106 | 0.084 | 1.877 | 0.489 | 0.131 | 0.424 | 0.476 | 0.363 | 0.007 |
| **o__Clostridiales** | 1.091 | 1.469 | 0.048 | 0.059 | 0.125 | 2.510 | 1.271 | 0.142 | 0.238 | 0.168 | 0.239 | 0.003 |
| **g__Oleibacter** | 0.010 | 0.000 | 0.617 | 3.484 | 1.977 | 0.005 | 0.000 | 0.000 | 0.010 | 0.382 | 0.610 | 0.056 |
| **f__Sphingomonadaceae** | 0.048 | 0.082 | 0.016 | 0.000 | 0.014 | 0.337 | 0.323 | 0.082 | 0.133 | 0.201 | 0.157 | 5.703 |
| **g__Sphingobium** | 0.048 | 0.010 | 0.000 | 0.000 | 0.014 | 0.011 | 0.018 | 0.000 | 0.000 | 0.114 | 0.033 | 6.528 |
| **g__Ruminococcus** | 1.206 | 1.939 | 0.011 | 0.000 | 0.097 | 1.476 | 1.057 | 0.028 | 0.248 | 0.295 | 0.297 | 0.007 |
| **g__Mycoplasma** | 1.457 | 1.694 | 0.005 | 0.000 | 0.070 | 0.575 | 0.039 | 0.230 | 0.395 | 0.958 | 0.173 | 0.007 |
| **g__Helicobacter** | 0.898 | 1.949 | 0.005 | 0.012 | 0.056 | 0.801 | 0.843 | 0.067 | 0.200 | 0.402 | 0.272 | 0.000 |
| **g__Thalassomonas** | 0.029 | 0.000 | 0.917 | 1.913 | 1.364 | 0.000 | 0.000 | 0.000 | 0.000 | 0.241 | 0.338 | 0.020 |
| **g__Ignatzschineria** | 0.010 | 0.010 | 0.445 | 0.614 | 0.668 | 0.469 | 0.000 | 0.004 | 0.024 | 0.657 | 1.756 | 0.000 |
| **o__Campylobacterales** | 0.145 | 0.000 | 1.572 | 1.193 | 1.587 | 0.000 | 0.000 | 0.000 | 0.000 | 0.034 | 0.008 | 0.000 |
| **f__Xanthomonadaceae** | 0.164 | 0.163 | 0.022 | 0.047 | 0.028 | 0.427 | 0.166 | 0.089 | 0.119 | 0.114 | 0.247 | 2.656 |
| **g__Brevundimonas** | 0.048 | 0.480 | 0.011 | 0.000 | 0.056 | 0.079 | 0.000 | 0.046 | 0.038 | 0.087 | 0.008 | 3.331 |
| **g__Micrococcus** | 0.531 | 0.255 | 0.150 | 0.000 | 0.097 | 0.337 | 0.695 | 1.297 | 0.176 | 0.201 | 0.091 | 0.007 |
| **g__Akkermansia** | 0.454 | 1.275 | 0.027 | 0.059 | 0.070 | 0.011 | 0.730 | 0.018 | 0.728 | 0.201 | 0.256 | 0.000 |
| **g__Sphingomonas** | 0.048 | 0.255 | 0.016 | 0.024 | 0.056 | 0.274 | 0.162 | 0.110 | 0.114 | 0.369 | 0.107 | 2.162 |
| **f__Rikenellaceae** | 0.811 | 1.531 | 0.032 | 0.000 | 0.084 | 0.026 | 0.127 | 0.050 | 0.276 | 0.583 | 0.050 | 0.000 |
| **g__Proteus** | 0.714 | 1.653 | 0.000 | 0.071 | 0.028 | 0.000 | 0.350 | 0.000 | 0.214 | 0.275 | 0.099 | 0.000 |
| **f__Pasteurellaceae** | 0.656 | 0.531 | 0.005 | 0.000 | 0.000 | 1.329 | 0.000 | 0.035 | 0.157 | 0.174 | 0.363 | 0.000 |
| **f__Flavobacteriaceae** | 0.048 | 0.010 | 0.220 | 0.555 | 0.278 | 0.037 | 0.000 | 0.032 | 0.062 | 0.623 | 1.335 | 0.023 |
| **g__Stenotrophomonas** | 0.087 | 0.184 | 0.016 | 0.012 | 0.000 | 0.032 | 0.179 | 0.032 | 0.029 | 0.007 | 0.041 | 2.561 |
| **g__Shimia** | 0.000 | 0.000 | 0.000 | 0.000 | 0.000 | 0.000 | 0.000 | 0.000 | 0.000 | 0.436 | 2.539 | 0.059 |
| **g__Oscillospira** | 0.984 | 1.061 | 0.000 | 0.035 | 0.014 | 0.432 | 0.000 | 0.004 | 0.105 | 0.261 | 0.132 | 0.003 |
| **o__Alteromonadales** | 0.010 | 0.010 | 0.976 | 0.638 | 0.738 | 0.005 | 0.013 | 0.046 | 0.195 | 0.208 | 0.190 | 0.000 |
| **g__Clostridium** | 1.013 | 0.847 | 0.000 | 0.047 | 0.000 | 0.701 | 0.227 | 0.000 | 0.010 | 0.020 | 0.008 | 0.000 |
| **c__Bacilli** | 0.338 | 0.133 | 0.032 | 0.047 | 0.125 | 0.395 | 0.459 | 0.277 | 0.500 | 0.382 | 0.140 | 0.026 |
| **o__Rhizobiales** | 0.135 | 0.031 | 0.011 | 0.012 | 0.042 | 0.322 | 0.201 | 0.188 | 0.229 | 0.275 | 0.074 | 1.320 |
| **g__Sutterella** | 0.444 | 1.306 | 0.000 | 0.000 | 0.014 | 0.501 | 0.232 | 0.135 | 0.091 | 0.020 | 0.082 | 0.010 |
| **o__Lactobacillales** | 0.241 | 0.296 | 0.022 | 0.106 | 0.139 | 0.917 | 0.354 | 0.082 | 0.119 | 0.194 | 0.313 | 0.013 |
| **g__Prevotella** | 0.232 | 0.276 | 0.070 | 0.012 | 0.028 | 1.624 | 0.000 | 0.103 | 0.110 | 0.174 | 0.041 | 0.000 |
| **g__Neisseria** | 0.434 | 0.418 | 0.016 | 0.000 | 0.000 | 1.339 | 0.026 | 0.092 | 0.076 | 0.168 | 0.041 | 0.000 |
| **g__Massilia** | 0.019 | 0.000 | 0.000 | 0.106 | 0.000 | 0.111 | 2.202 | 0.035 | 0.095 | 0.000 | 0.000 | 0.000 |
| **g__Fusobacterium** | 0.077 | 0.163 | 0.000 | 0.000 | 0.028 | 1.735 | 0.153 | 0.174 | 0.000 | 0.201 | 0.000 | 0.000 |
| **o__Chloroflexales** | 0.425 | 0.020 | 0.000 | 0.000 | 0.111 | 0.000 | 0.502 | 0.000 | 0.743 | 0.623 | 0.017 | 0.003 |
| **g__[Prevotella]** | 0.376 | 0.551 | 0.000 | 0.000 | 0.000 | 0.780 | 0.310 | 0.014 | 0.129 | 0.248 | 0.017 | 0.000 |
| **g__Hydrogenophilus** | 0.000 | 0.000 | 0.081 | 0.000 | 0.000 | 1.112 | 0.310 | 0.401 | 0.214 | 0.134 | 0.140 | 0.000 |
| **g__Chryseobacterium** | 0.116 | 0.000 | 0.005 | 0.000 | 0.028 | 0.627 | 0.048 | 0.007 | 0.043 | 0.395 | 0.008 | 1.101 |
| **g__Magnetospirillum** | 0.019 | 0.010 | 0.022 | 0.000 | 0.000 | 0.000 | 0.000 | 0.000 | 0.038 | 0.027 | 0.181 | 2.060 |
| **g__Brevibacterium** | 0.328 | 0.010 | 0.022 | 0.000 | 0.042 | 0.021 | 0.000 | 0.078 | 0.786 | 0.798 | 0.115 | 0.039 |
| **g__Odoribacter** | 0.463 | 0.735 | 0.000 | 0.000 | 0.014 | 0.638 | 0.000 | 0.085 | 0.000 | 0.188 | 0.025 | 0.000 |
| **g__Allobaculum** | 0.502 | 1.020 | 0.005 | 0.095 | 0.056 | 0.042 | 0.013 | 0.032 | 0.105 | 0.087 | 0.140 | 0.000 |
| **g__Lactobacillus** | 0.077 | 0.225 | 0.022 | 0.201 | 0.084 | 0.332 | 0.022 | 0.018 | 0.167 | 0.657 | 0.289 | 0.000 |
| **g__Alistipes** | 0.347 | 0.837 | 0.032 | 0.024 | 0.042 | 0.005 | 0.018 | 0.035 | 0.205 | 0.362 | 0.132 | 0.013 |
| **o__Burkholderiales** | 0.135 | 0.622 | 0.016 | 0.012 | 0.014 | 0.237 | 0.406 | 0.032 | 0.052 | 0.040 | 0.050 | 0.328 |
| **g__[Clostridium]** | 0.357 | 0.184 | 0.022 | 0.012 | 0.042 | 0.169 | 0.385 | 0.032 | 0.048 | 0.416 | 0.074 | 0.000 |
| **g__Tenacibaculum** | 0.010 | 0.000 | 0.236 | 0.933 | 0.390 | 0.000 | 0.000 | 0.000 | 0.000 | 0.094 | 0.025 | 0.000 |
| **g__Mucispirillum** | 0.280 | 0.582 | 0.000 | 0.000 | 0.000 | 0.185 | 0.258 | 0.057 | 0.186 | 0.087 | 0.000 | 0.000 |
| **g__YRC22** | 0.164 | 0.174 | 0.000 | 0.000 | 0.000 | 1.086 | 0.000 | 0.007 | 0.114 | 0.040 | 0.000 | 0.000 |
| **g__Clostridium** | 0.357 | 0.102 | 0.000 | 0.035 | 0.139 | 0.000 | 0.000 | 0.014 | 0.219 | 0.597 | 0.017 | 0.000 |
| **g__Desulfovibrio** | 0.376 | 0.888 | 0.005 | 0.000 | 0.014 | 0.074 | 0.009 | 0.000 | 0.000 | 0.013 | 0.066 | 0.000 |
| **f__Peptostreptococcaceae** | 0.280 | 0.235 | 0.005 | 0.012 | 0.056 | 0.100 | 0.450 | 0.025 | 0.019 | 0.248 | 0.000 | 0.000 |
| **g__Erythrobacter** | 0.000 | 0.000 | 0.022 | 0.130 | 0.000 | 0.000 | 0.000 | 0.000 | 0.000 | 0.449 | 0.750 | 0.007 |
| **f__Oleiphilaceae** | 0.000 | 0.000 | 0.000 | 0.071 | 0.000 | 0.000 | 0.000 | 0.000 | 0.000 | 0.148 | 1.080 | 0.043 |
| **o__RF39** | 0.212 | 0.296 | 0.016 | 0.024 | 0.070 | 0.195 | 0.398 | 0.060 | 0.010 | 0.040 | 0.008 | 0.000 |
| **g__Roseomonas** | 0.010 | 0.071 | 0.000 | 0.012 | 0.000 | 0.158 | 0.000 | 0.060 | 0.000 | 0.074 | 0.017 | 0.924 |
| **f__Oceanospirillaceae** | 0.010 | 0.010 | 0.134 | 0.650 | 0.348 | 0.000 | 0.000 | 0.000 | 0.000 | 0.054 | 0.107 | 0.000 |
| **f__Rhodocyclaceae** | 0.019 | 0.020 | 0.113 | 0.000 | 0.042 | 0.216 | 0.227 | 0.542 | 0.029 | 0.060 | 0.025 | 0.000 |
| **f__wb1_P06** | 0.068 | 0.061 | 0.032 | 0.000 | 0.195 | 0.000 | 0.454 | 0.000 | 0.119 | 0.308 | 0.025 | 0.000 |
| **o__Flavobacteriales** | 0.077 | 0.000 | 0.048 | 0.071 | 0.056 | 0.158 | 0.048 | 0.011 | 0.062 | 0.389 | 0.190 | 0.147 |
| **f__Staphylococcaceae** | 0.193 | 0.020 | 0.011 | 0.012 | 0.056 | 0.000 | 0.179 | 0.163 | 0.157 | 0.456 | 0.008 | 0.000 |
| **g__Glaciecola** | 0.000 | 0.000 | 0.241 | 0.532 | 0.348 | 0.000 | 0.000 | 0.000 | 0.000 | 0.080 | 0.033 | 0.000 |
| **f__Intrasporangiaceae** | 0.154 | 0.031 | 0.059 | 0.000 | 0.028 | 0.000 | 0.066 | 0.082 | 0.348 | 0.402 | 0.058 | 0.007 |
| **g__Veillonella** | 0.174 | 0.071 | 0.000 | 0.012 | 0.000 | 0.807 | 0.013 | 0.011 | 0.000 | 0.054 | 0.000 | 0.003 |
| **o__Sphingomonadales** | 0.019 | 0.020 | 0.016 | 0.012 | 0.014 | 0.011 | 0.061 | 0.014 | 0.033 | 0.060 | 0.017 | 0.835 |
| **p__Cyanobacteria** | 0.029 | 0.010 | 0.129 | 0.012 | 0.042 | 0.169 | 0.197 | 0.344 | 0.114 | 0.020 | 0.025 | 0.007 |
| **g__Shigella** | 0.232 | 0.174 | 0.000 | 0.012 | 0.014 | 0.116 | 0.376 | 0.018 | 0.029 | 0.034 | 0.050 | 0.000 |
| **c__BD1-5** | 0.000 | 0.000 | 0.225 | 0.366 | 0.390 | 0.000 | 0.000 | 0.000 | 0.000 | 0.020 | 0.033 | 0.003 |
| **f__Colwelliaceae** | 0.000 | 0.000 | 0.177 | 0.520 | 0.223 | 0.005 | 0.000 | 0.000 | 0.000 | 0.027 | 0.074 | 0.007 |
| **g__Leptotrichia** | 0.029 | 0.163 | 0.005 | 0.000 | 0.000 | 0.707 | 0.000 | 0.004 | 0.057 | 0.020 | 0.000 | 0.000 |
| **g__AF12** | 0.328 | 0.541 | 0.000 | 0.000 | 0.000 | 0.000 | 0.000 | 0.000 | 0.000 | 0.040 | 0.050 | 0.000 |
| **g__Burkholderia** | 0.203 | 0.459 | 0.011 | 0.000 | 0.014 | 0.090 | 0.083 | 0.004 | 0.010 | 0.060 | 0.000 | 0.000 |
| **o__Streptophyta** | 0.000 | 0.020 | 0.762 | 0.000 | 0.000 | 0.000 | 0.000 | 0.018 | 0.119 | 0.000 | 0.008 | 0.000 |
| **f__Cryomorphaceae** | 0.010 | 0.000 | 0.027 | 0.024 | 0.014 | 0.000 | 0.000 | 0.000 | 0.000 | 0.315 | 0.486 | 0.030 |
| **g__Marinomonas** | 0.019 | 0.010 | 0.145 | 0.413 | 0.251 | 0.000 | 0.000 | 0.000 | 0.000 | 0.007 | 0.033 | 0.003 |
| **f__Burkholderiaceae** | 0.174 | 0.408 | 0.000 | 0.000 | 0.000 | 0.090 | 0.131 | 0.000 | 0.005 | 0.027 | 0.033 | 0.000 |
| **o__Oceanospirillales** | 0.000 | 0.000 | 0.070 | 0.461 | 0.097 | 0.000 | 0.000 | 0.011 | 0.000 | 0.054 | 0.157 | 0.007 |
| **f__Neisseriaceae** | 0.058 | 0.102 | 0.038 | 0.000 | 0.000 | 0.432 | 0.013 | 0.128 | 0.029 | 0.020 | 0.017 | 0.000 |
| **f__Lactobacillaceae** | 0.058 | 0.061 | 0.016 | 0.000 | 0.014 | 0.253 | 0.000 | 0.011 | 0.076 | 0.342 | 0.000 | 0.000 |
| **c__Clostridia** | 0.145 | 0.245 | 0.000 | 0.012 | 0.000 | 0.195 | 0.061 | 0.032 | 0.038 | 0.047 | 0.033 | 0.007 |
| **g__Paraprevotella** | 0.222 | 0.174 | 0.000 | 0.000 | 0.000 | 0.016 | 0.000 | 0.000 | 0.100 | 0.282 | 0.000 | 0.010 |
| **g__Campylobacter** | 0.019 | 0.000 | 0.000 | 0.000 | 0.000 | 0.780 | 0.000 | 0.000 | 0.000 | 0.000 | 0.000 | 0.000 |
| **f__[Weeksellaceae]** | 0.029 | 0.010 | 0.000 | 0.000 | 0.028 | 0.227 | 0.013 | 0.018 | 0.038 | 0.141 | 0.017 | 0.269 |
| **g__Saprospira** | 0.000 | 0.000 | 0.000 | 0.000 | 0.000 | 0.000 | 0.000 | 0.000 | 0.000 | 0.248 | 0.519 | 0.013 |
| **f__Porphyromonadaceae** | 0.126 | 0.082 | 0.005 | 0.035 | 0.097 | 0.079 | 0.000 | 0.021 | 0.010 | 0.261 | 0.058 | 0.000 |
| **g__Haemophilus** | 0.097 | 0.092 | 0.011 | 0.000 | 0.000 | 0.401 | 0.000 | 0.018 | 0.029 | 0.060 | 0.025 | 0.000 |
| **g__Brachybacterium** | 0.135 | 0.031 | 0.016 | 0.024 | 0.056 | 0.000 | 0.000 | 0.294 | 0.000 | 0.174 | 0.000 | 0.000 |
| **g__Acidovorax** | 0.039 | 0.010 | 0.016 | 0.000 | 0.000 | 0.047 | 0.489 | 0.004 | 0.043 | 0.027 | 0.041 | 0.000 |
| **g__SGUS912** | 0.106 | 0.000 | 0.000 | 0.000 | 0.042 | 0.000 | 0.000 | 0.000 | 0.100 | 0.416 | 0.017 | 0.016 |
| **f__Oxalobacteraceae** | 0.000 | 0.000 | 0.011 | 0.012 | 0.000 | 0.121 | 0.332 | 0.007 | 0.033 | 0.000 | 0.008 | 0.167 |
| **g__Actinomyces** | 0.097 | 0.051 | 0.075 | 0.000 | 0.000 | 0.279 | 0.035 | 0.021 | 0.076 | 0.027 | 0.017 | 0.000 |
| **g__Macrococcus** | 0.097 | 0.010 | 0.005 | 0.000 | 0.000 | 0.000 | 0.184 | 0.199 | 0.029 | 0.148 | 0.000 | 0.000 |
| **g__Neptunomonas** | 0.000 | 0.000 | 0.075 | 0.130 | 0.292 | 0.000 | 0.000 | 0.000 | 0.000 | 0.060 | 0.107 | 0.003 |
| **f__Erythrobacteraceae** | 0.000 | 0.000 | 0.005 | 0.024 | 0.042 | 0.005 | 0.000 | 0.082 | 0.071 | 0.194 | 0.239 | 0.003 |
| **f__Rhizobiaceae** | 0.000 | 0.000 | 0.000 | 0.000 | 0.000 | 0.000 | 0.262 | 0.014 | 0.000 | 0.121 | 0.000 | 0.259 |
| **g__Perlucidibaca** | 0.106 | 0.000 | 0.000 | 0.000 | 0.000 | 0.148 | 0.000 | 0.025 | 0.267 | 0.107 | 0.000 | 0.000 |
| **o__BPC015** | 0.058 | 0.000 | 0.000 | 0.000 | 0.070 | 0.000 | 0.000 | 0.000 | 0.214 | 0.288 | 0.017 | 0.000 |
| **g__Methylobacterium** | 0.010 | 0.020 | 0.000 | 0.000 | 0.028 | 0.195 | 0.000 | 0.099 | 0.000 | 0.101 | 0.008 | 0.170 |
| **f__Bacillaceae** | 0.077 | 0.296 | 0.011 | 0.000 | 0.028 | 0.011 | 0.009 | 0.043 | 0.119 | 0.007 | 0.017 | 0.003 |
| **g__Methanobrevibacter** | 0.000 | 0.000 | 0.000 | 0.000 | 0.000 | 0.000 | 0.306 | 0.035 | 0.000 | 0.194 | 0.074 | 0.000 |
| **g__Paracoccus** | 0.019 | 0.041 | 0.005 | 0.012 | 0.000 | 0.000 | 0.131 | 0.050 | 0.133 | 0.114 | 0.099 | 0.003 |
| **g__Enterococcus** | 0.097 | 0.031 | 0.000 | 0.024 | 0.000 | 0.142 | 0.000 | 0.046 | 0.038 | 0.194 | 0.033 | 0.000 |
| **f__Alcaligenaceae** | 0.126 | 0.225 | 0.000 | 0.000 | 0.000 | 0.090 | 0.022 | 0.046 | 0.005 | 0.007 | 0.017 | 0.069 |
| **f__Erysipelotrichaceae** | 0.087 | 0.225 | 0.005 | 0.000 | 0.000 | 0.037 | 0.048 | 0.004 | 0.062 | 0.027 | 0.091 | 0.000 |
| **c__Oscillatoriophycideae** | 0.048 | 0.000 | 0.005 | 0.000 | 0.014 | 0.174 | 0.105 | 0.149 | 0.048 | 0.027 | 0.000 | 0.000 |
| **g__Coprococcus** | 0.280 | 0.184 | 0.000 | 0.000 | 0.000 | 0.037 | 0.000 | 0.004 | 0.010 | 0.000 | 0.050 | 0.000 |
| **g__Chroococcidiopsis** | 0.087 | 0.000 | 0.000 | 0.000 | 0.000 | 0.000 | 0.000 | 0.032 | 0.095 | 0.328 | 0.008 | 0.000 |
| **g__Halomonas** | 0.039 | 0.000 | 0.011 | 0.035 | 0.000 | 0.000 | 0.000 | 0.188 | 0.186 | 0.080 | 0.008 | 0.000 |
| **p__Actinobacteria** | 0.039 | 0.031 | 0.048 | 0.000 | 0.000 | 0.000 | 0.092 | 0.025 | 0.152 | 0.148 | 0.008 | 0.000 |
| **f__TK06** | 0.039 | 0.000 | 0.000 | 0.000 | 0.014 | 0.000 | 0.000 | 0.000 | 0.076 | 0.382 | 0.008 | 0.000 |
| **f__Dermabacteraceae** | 0.068 | 0.010 | 0.005 | 0.012 | 0.000 | 0.000 | 0.000 | 0.390 | 0.000 | 0.034 | 0.000 | 0.000 |
| **c__SAR202** | 0.087 | 0.010 | 0.000 | 0.000 | 0.056 | 0.000 | 0.000 | 0.000 | 0.143 | 0.215 | 0.000 | 0.000 |
| **g__Treponema** | 0.039 | 0.051 | 0.000 | 0.000 | 0.000 | 0.279 | 0.074 | 0.004 | 0.000 | 0.027 | 0.025 | 0.000 |
| **g__Moryella** | 0.019 | 0.000 | 0.000 | 0.000 | 0.000 | 0.443 | 0.000 | 0.000 | 0.000 | 0.000 | 0.000 | 0.000 |
| **g__Oleispira** | 0.000 | 0.000 | 0.048 | 0.177 | 0.181 | 0.000 | 0.000 | 0.000 | 0.000 | 0.054 | 0.000 | 0.000 |
| **f__Ulvophyceae** | 0.029 | 0.000 | 0.000 | 0.000 | 0.042 | 0.000 | 0.000 | 0.007 | 0.005 | 0.315 | 0.058 | 0.000 |
| **g__5-7N15** | 0.000 | 0.000 | 0.005 | 0.000 | 0.014 | 0.190 | 0.240 | 0.000 | 0.000 | 0.000 | 0.000 | 0.000 |
| **f__[Mogibacteriaceae]** | 0.116 | 0.235 | 0.000 | 0.000 | 0.028 | 0.058 | 0.000 | 0.000 | 0.005 | 0.000 | 0.008 | 0.000 |
| **f__Pseudoalteromonadaceae** | 0.010 | 0.020 | 0.156 | 0.142 | 0.111 | 0.000 | 0.000 | 0.000 | 0.000 | 0.000 | 0.000 | 0.000 |
| **f__Caulobacteraceae** | 0.010 | 0.020 | 0.000 | 0.000 | 0.028 | 0.069 | 0.000 | 0.028 | 0.076 | 0.127 | 0.000 | 0.079 |
| **g__Reinekea** | 0.000 | 0.000 | 0.016 | 0.106 | 0.042 | 0.000 | 0.000 | 0.000 | 0.000 | 0.188 | 0.058 | 0.020 |
| **g__Vibrio** | 0.010 | 0.041 | 0.166 | 0.106 | 0.097 | 0.000 | 0.000 | 0.000 | 0.000 | 0.000 | 0.000 | 0.003 |
| **g__Parabacteroides** | 0.126 | 0.204 | 0.000 | 0.000 | 0.000 | 0.079 | 0.000 | 0.000 | 0.000 | 0.000 | 0.008 | 0.000 |
| **f__Rhodospirillaceae** | 0.019 | 0.010 | 0.011 | 0.000 | 0.000 | 0.243 | 0.000 | 0.004 | 0.057 | 0.060 | 0.000 | 0.007 |
| **g__Mycobacterium** | 0.106 | 0.020 | 0.005 | 0.000 | 0.014 | 0.000 | 0.000 | 0.007 | 0.095 | 0.121 | 0.041 | 0.000 |
| **f__Verrucomicrobiaceae** | 0.000 | 0.112 | 0.011 | 0.000 | 0.000 | 0.000 | 0.048 | 0.025 | 0.148 | 0.013 | 0.033 | 0.000 |
| **c__Epsilonproteobacteria** | 0.000 | 0.000 | 0.102 | 0.106 | 0.167 | 0.000 | 0.000 | 0.000 | 0.000 | 0.007 | 0.000 | 0.000 |
| **g__CF231** | 0.000 | 0.000 | 0.011 | 0.000 | 0.000 | 0.364 | 0.000 | 0.000 | 0.000 | 0.007 | 0.000 | 0.000 |
| **g__Agrobacterium** | 0.000 | 0.000 | 0.000 | 0.000 | 0.000 | 0.000 | 0.009 | 0.004 | 0.000 | 0.000 | 0.000 | 0.364 |
| **g__Macellibacteroides** | 0.000 | 0.000 | 0.000 | 0.000 | 0.000 | 0.359 | 0.000 | 0.000 | 0.000 | 0.013 | 0.000 | 0.000 |
| **f__Acetobacteraceae** | 0.048 | 0.061 | 0.005 | 0.000 | 0.000 | 0.026 | 0.000 | 0.000 | 0.000 | 0.000 | 0.091 | 0.131 |
| **g__Kytococcus** | 0.087 | 0.010 | 0.016 | 0.000 | 0.000 | 0.000 | 0.000 | 0.014 | 0.119 | 0.114 | 0.000 | 0.000 |
| **g__Anaerococcus** | 0.068 | 0.000 | 0.005 | 0.000 | 0.014 | 0.000 | 0.000 | 0.191 | 0.024 | 0.047 | 0.000 | 0.000 |
| **g__Truepera** | 0.019 | 0.000 | 0.000 | 0.000 | 0.028 | 0.000 | 0.000 | 0.000 | 0.024 | 0.268 | 0.008 | 0.000 |
| **g__Butyricicoccus** | 0.029 | 0.112 | 0.000 | 0.000 | 0.000 | 0.000 | 0.201 | 0.000 | 0.000 | 0.000 | 0.000 | 0.000 |
| **g__Blastococcus** | 0.000 | 0.000 | 0.000 | 0.000 | 0.000 | 0.279 | 0.000 | 0.000 | 0.062 | 0.000 | 0.000 | 0.000 |
| **g__Capnocytophaga** | 0.019 | 0.000 | 0.005 | 0.000 | 0.000 | 0.316 | 0.000 | 0.000 | 0.000 | 0.000 | 0.000 | 0.000 |
| **g__Spirochaeta** | 0.000 | 0.000 | 0.000 | 0.000 | 0.014 | 0.000 | 0.105 | 0.071 | 0.000 | 0.148 | 0.000 | 0.000 |
| **f__Microbacteriaceae** | 0.000 | 0.000 | 0.000 | 0.000 | 0.014 | 0.000 | 0.135 | 0.156 | 0.005 | 0.020 | 0.000 | 0.000 |
| **f__Veillonellaceae** | 0.019 | 0.010 | 0.000 | 0.000 | 0.014 | 0.200 | 0.000 | 0.025 | 0.000 | 0.027 | 0.033 | 0.000 |
| **c__Deltaproteobacteria** | 0.010 | 0.031 | 0.005 | 0.024 | 0.000 | 0.021 | 0.000 | 0.004 | 0.010 | 0.134 | 0.082 | 0.000 |
| **g__Arsenophonus** | 0.000 | 0.000 | 0.000 | 0.000 | 0.000 | 0.000 | 0.000 | 0.000 | 0.319 | 0.000 | 0.000 | 0.000 |
| **f__Planococcaceae** | 0.000 | 0.031 | 0.000 | 0.000 | 0.000 | 0.211 | 0.004 | 0.021 | 0.014 | 0.000 | 0.033 | 0.000 |
| **g__Bacteriovorax** | 0.000 | 0.000 | 0.016 | 0.177 | 0.056 | 0.000 | 0.000 | 0.000 | 0.000 | 0.000 | 0.058 | 0.000 |
| **g__N09** | 0.000 | 0.031 | 0.000 | 0.000 | 0.000 | 0.274 | 0.000 | 0.000 | 0.000 | 0.000 | 0.000 | 0.000 |
| **g__Halalkalicoccus** | 0.077 | 0.000 | 0.027 | 0.000 | 0.014 | 0.000 | 0.000 | 0.025 | 0.005 | 0.148 | 0.000 | 0.000 |
| **c__EC214** | 0.000 | 0.000 | 0.000 | 0.012 | 0.000 | 0.000 | 0.000 | 0.000 | 0.214 | 0.027 | 0.000 | 0.039 |
| **g__Rhizobium** | 0.000 | 0.010 | 0.000 | 0.000 | 0.000 | 0.000 | 0.052 | 0.000 | 0.000 | 0.000 | 0.000 | 0.229 |
| **o__TK10** | 0.029 | 0.000 | 0.000 | 0.000 | 0.028 | 0.148 | 0.000 | 0.000 | 0.019 | 0.067 | 0.000 | 0.000 |
| **g__Cardiobacterium** | 0.029 | 0.000 | 0.000 | 0.000 | 0.000 | 0.000 | 0.000 | 0.018 | 0.057 | 0.168 | 0.000 | 0.000 |
| **f__Desulfobacteraceae** | 0.000 | 0.000 | 0.000 | 0.000 | 0.000 | 0.269 | 0.000 | 0.000 | 0.000 | 0.000 | 0.000 | 0.000 |
| **o__YS2** | 0.058 | 0.010 | 0.000 | 0.024 | 0.000 | 0.121 | 0.000 | 0.000 | 0.048 | 0.000 | 0.008 | 0.000 |
| **g__Dorea** | 0.048 | 0.031 | 0.000 | 0.000 | 0.000 | 0.190 | 0.000 | 0.000 | 0.000 | 0.000 | 0.000 | 0.000 |
| **g__Turicibacter** | 0.048 | 0.102 | 0.022 | 0.024 | 0.000 | 0.058 | 0.004 | 0.011 | 0.000 | 0.000 | 0.000 | 0.000 |
| **g__Cupriavidus** | 0.000 | 0.000 | 0.000 | 0.000 | 0.000 | 0.000 | 0.000 | 0.000 | 0.000 | 0.000 | 0.000 | 0.265 |
| **f__Bacteriovoracaceae** | 0.000 | 0.000 | 0.011 | 0.142 | 0.056 | 0.000 | 0.000 | 0.000 | 0.000 | 0.007 | 0.050 | 0.000 |
| **g__Xanthobacter** | 0.000 | 0.000 | 0.000 | 0.000 | 0.000 | 0.000 | 0.000 | 0.000 | 0.005 | 0.047 | 0.008 | 0.200 |
| **g__Bacillus** | 0.000 | 0.092 | 0.054 | 0.000 | 0.000 | 0.005 | 0.000 | 0.018 | 0.091 | 0.000 | 0.000 | 0.000 |
| **g__Skermanella** | 0.000 | 0.000 | 0.000 | 0.000 | 0.014 | 0.000 | 0.000 | 0.000 | 0.000 | 0.228 | 0.017 | 0.000 |
| **c__Gemm-2** | 0.039 | 0.000 | 0.000 | 0.000 | 0.014 | 0.000 | 0.000 | 0.000 | 0.114 | 0.074 | 0.017 | 0.000 |
| **g__Wohlfahrtiimonas** | 0.000 | 0.000 | 0.005 | 0.024 | 0.014 | 0.000 | 0.000 | 0.000 | 0.000 | 0.074 | 0.140 | 0.000 |
| **g__Atopostipes** | 0.029 | 0.061 | 0.000 | 0.012 | 0.070 | 0.000 | 0.000 | 0.057 | 0.000 | 0.020 | 0.008 | 0.000 |
| **o__RF32** | 0.164 | 0.092 | 0.000 | 0.000 | 0.000 | 0.000 | 0.000 | 0.000 | 0.000 | 0.000 | 0.000 | 0.000 |
| **g__Clostridium** | 0.048 | 0.143 | 0.000 | 0.000 | 0.000 | 0.053 | 0.000 | 0.007 | 0.005 | 0.000 | 0.000 | 0.000 |
| **g__Deinococcus** | 0.019 | 0.000 | 0.000 | 0.000 | 0.000 | 0.000 | 0.000 | 0.021 | 0.200 | 0.013 | 0.000 | 0.000 |
| **g__Amphritea** | 0.000 | 0.000 | 0.059 | 0.071 | 0.084 | 0.000 | 0.000 | 0.000 | 0.000 | 0.007 | 0.025 | 0.003 |
| **g__Caulobacter** | 0.000 | 0.000 | 0.000 | 0.000 | 0.000 | 0.000 | 0.000 | 0.000 | 0.000 | 0.000 | 0.000 | 0.246 |
| **g__Methylopila** | 0.000 | 0.000 | 0.000 | 0.000 | 0.014 | 0.000 | 0.000 | 0.000 | 0.000 | 0.000 | 0.000 | 0.229 |
| **f__Bradyrhizobiaceae** | 0.000 | 0.000 | 0.032 | 0.000 | 0.000 | 0.000 | 0.000 | 0.135 | 0.024 | 0.034 | 0.017 | 0.000 |
| **p__AncK6** | 0.000 | 0.000 | 0.000 | 0.000 | 0.000 | 0.000 | 0.000 | 0.000 | 0.010 | 0.228 | 0.000 | 0.000 |
| **g__Bifidobacterium** | 0.000 | 0.000 | 0.000 | 0.024 | 0.000 | 0.142 | 0.000 | 0.050 | 0.000 | 0.000 | 0.008 | 0.003 |
| **f__Sphingobacteriaceae** | 0.000 | 0.031 | 0.000 | 0.000 | 0.000 | 0.142 | 0.000 | 0.000 | 0.000 | 0.013 | 0.033 | 0.007 |
| **g__Nautella** | 0.000 | 0.000 | 0.000 | 0.012 | 0.000 | 0.000 | 0.000 | 0.000 | 0.000 | 0.020 | 0.190 | 0.003 |
| **o__TK18** | 0.000 | 0.000 | 0.005 | 0.000 | 0.014 | 0.000 | 0.000 | 0.000 | 0.091 | 0.114 | 0.000 | 0.000 |
| **f__Methylobacteriaceae** | 0.010 | 0.000 | 0.000 | 0.000 | 0.028 | 0.079 | 0.000 | 0.025 | 0.000 | 0.013 | 0.000 | 0.059 |
| **c__Synechococcophycideae** | 0.000 | 0.010 | 0.000 | 0.000 | 0.000 | 0.174 | 0.000 | 0.000 | 0.000 | 0.020 | 0.008 | 0.000 |
| **g__Rubellimicrobium** | 0.010 | 0.000 | 0.000 | 0.000 | 0.000 | 0.000 | 0.000 | 0.000 | 0.000 | 0.194 | 0.008 | 0.000 |
| **g__Propionibacterium** | 0.000 | 0.000 | 0.005 | 0.000 | 0.000 | 0.132 | 0.000 | 0.018 | 0.000 | 0.007 | 0.050 | 0.000 |
| **g__Tropicibacter** | 0.000 | 0.000 | 0.000 | 0.000 | 0.000 | 0.000 | 0.000 | 0.000 | 0.000 | 0.020 | 0.190 | 0.000 |
| **f__Helicobacteraceae** | 0.097 | 0.051 | 0.005 | 0.000 | 0.028 | 0.005 | 0.013 | 0.000 | 0.000 | 0.000 | 0.008 | 0.000 |
| **g__Escherichia** | 0.029 | 0.000 | 0.000 | 0.000 | 0.000 | 0.000 | 0.162 | 0.004 | 0.000 | 0.007 | 0.000 | 0.000 |
| **g__Thermus** | 0.000 | 0.000 | 0.000 | 0.024 | 0.028 | 0.016 | 0.000 | 0.000 | 0.062 | 0.000 | 0.066 | 0.000 |
| **g__Mucilaginibacter** | 0.000 | 0.000 | 0.000 | 0.000 | 0.014 | 0.132 | 0.000 | 0.000 | 0.000 | 0.027 | 0.008 | 0.007 |
| **g__Arthrobacter** | 0.077 | 0.000 | 0.000 | 0.000 | 0.000 | 0.053 | 0.013 | 0.011 | 0.000 | 0.034 | 0.000 | 0.000 |
| **f__Vibrionaceae** | 0.000 | 0.000 | 0.070 | 0.035 | 0.070 | 0.000 | 0.000 | 0.000 | 0.005 | 0.007 | 0.000 | 0.000 |
| **g__Weissella** | 0.000 | 0.000 | 0.000 | 0.000 | 0.000 | 0.179 | 0.000 | 0.000 | 0.000 | 0.000 | 0.000 | 0.000 |
| **f__Enterococcaceae** | 0.019 | 0.000 | 0.000 | 0.012 | 0.014 | 0.032 | 0.000 | 0.004 | 0.024 | 0.074 | 0.000 | 0.000 |
| **g__Ponticaulis** | 0.000 | 0.000 | 0.000 | 0.024 | 0.000 | 0.000 | 0.000 | 0.000 | 0.000 | 0.094 | 0.050 | 0.010 |
| **g__Dysgonomonas** | 0.000 | 0.000 | 0.005 | 0.035 | 0.056 | 0.058 | 0.000 | 0.000 | 0.000 | 0.020 | 0.000 | 0.000 |
| **o__Rhodospirillales** | 0.000 | 0.000 | 0.000 | 0.000 | 0.000 | 0.053 | 0.000 | 0.014 | 0.033 | 0.060 | 0.008 | 0.003 |
| **g__Fimbriimonas** | 0.000 | 0.000 | 0.011 | 0.000 | 0.000 | 0.000 | 0.000 | 0.082 | 0.057 | 0.013 | 0.008 | 0.000 |
| **g__Coprobacillus** | 0.019 | 0.010 | 0.000 | 0.000 | 0.000 | 0.000 | 0.127 | 0.000 | 0.014 | 0.000 | 0.000 | 0.000 |
| **f__Dermatophilaceae** | 0.000 | 0.000 | 0.000 | 0.000 | 0.000 | 0.148 | 0.000 | 0.000 | 0.014 | 0.000 | 0.008 | 0.000 |
| **f__Spirochaetaceae** | 0.010 | 0.031 | 0.000 | 0.000 | 0.000 | 0.026 | 0.096 | 0.000 | 0.000 | 0.007 | 0.000 | 0.000 |
| **g__Devosia** | 0.000 | 0.000 | 0.000 | 0.000 | 0.000 | 0.169 | 0.000 | 0.000 | 0.000 | 0.000 | 0.000 | 0.000 |
| **p__SR1** | 0.019 | 0.061 | 0.000 | 0.000 | 0.000 | 0.011 | 0.000 | 0.004 | 0.000 | 0.074 | 0.000 | 0.000 |
| **o__Stramenopiles** | 0.000 | 0.010 | 0.000 | 0.000 | 0.000 | 0.000 | 0.153 | 0.000 | 0.000 | 0.000 | 0.000 | 0.000 |
| **p__Chloroflexi** | 0.039 | 0.000 | 0.000 | 0.000 | 0.000 | 0.011 | 0.048 | 0.004 | 0.033 | 0.020 | 0.008 | 0.000 |
| **g__Fluviicola** | 0.000 | 0.000 | 0.022 | 0.000 | 0.028 | 0.000 | 0.000 | 0.000 | 0.000 | 0.040 | 0.066 | 0.007 |
| **o__Solibacterales** | 0.000 | 0.000 | 0.000 | 0.000 | 0.000 | 0.000 | 0.162 | 0.000 | 0.000 | 0.000 | 0.000 | 0.000 |
| **g__Finegoldia** | 0.000 | 0.000 | 0.005 | 0.000 | 0.000 | 0.148 | 0.000 | 0.000 | 0.000 | 0.000 | 0.008 | 0.000 |
| **g__Zoogloea** | 0.000 | 0.000 | 0.000 | 0.000 | 0.000 | 0.153 | 0.000 | 0.004 | 0.000 | 0.000 | 0.000 | 0.000 |
| **g__Candidatus_Portiera** | 0.029 | 0.000 | 0.005 | 0.024 | 0.042 | 0.000 | 0.000 | 0.004 | 0.005 | 0.007 | 0.041 | 0.000 |
| **f__Xenococcaceae** | 0.000 | 0.000 | 0.000 | 0.000 | 0.000 | 0.000 | 0.092 | 0.032 | 0.014 | 0.013 | 0.000 | 0.000 |
| **g__Enterobacter** | 0.039 | 0.051 | 0.005 | 0.000 | 0.000 | 0.011 | 0.013 | 0.004 | 0.005 | 0.020 | 0.000 | 0.003 |
| **g__Morganella** | 0.058 | 0.000 | 0.000 | 0.000 | 0.000 | 0.000 | 0.052 | 0.000 | 0.000 | 0.007 | 0.033 | 0.000 |
| **g__Oceanimonas** | 0.000 | 0.000 | 0.000 | 0.035 | 0.014 | 0.000 | 0.000 | 0.000 | 0.000 | 0.000 | 0.099 | 0.000 |
| **g__Rheinheimera** | 0.000 | 0.000 | 0.005 | 0.000 | 0.000 | 0.000 | 0.135 | 0.000 | 0.000 | 0.000 | 0.000 | 0.007 |
| **g__Flexispira** | 0.116 | 0.010 | 0.000 | 0.000 | 0.000 | 0.016 | 0.004 | 0.000 | 0.000 | 0.000 | 0.000 | 0.000 |
| **g__Neptuniibacter** | 0.000 | 0.010 | 0.054 | 0.012 | 0.070 | 0.000 | 0.000 | 0.000 | 0.000 | 0.000 | 0.000 | 0.000 |
| **g__Flexibacter** | 0.000 | 0.000 | 0.011 | 0.106 | 0.014 | 0.000 | 0.000 | 0.000 | 0.000 | 0.013 | 0.000 | 0.000 |
| **g__Gemella** | 0.019 | 0.020 | 0.000 | 0.000 | 0.000 | 0.021 | 0.000 | 0.018 | 0.000 | 0.020 | 0.008 | 0.036 |
| **f__WCHB1-25** | 0.000 | 0.000 | 0.000 | 0.000 | 0.000 | 0.142 | 0.000 | 0.000 | 0.000 | 0.000 | 0.000 | 0.000 |
| **c__Mollicutes** | 0.010 | 0.031 | 0.005 | 0.000 | 0.014 | 0.016 | 0.057 | 0.000 | 0.005 | 0.000 | 0.000 | 0.003 |
| **g__Achromobacter** | 0.058 | 0.082 | 0.000 | 0.000 | 0.000 | 0.000 | 0.000 | 0.000 | 0.000 | 0.000 | 0.000 | 0.000 |
| **p__PAUC34f** | 0.000 | 0.051 | 0.000 | 0.000 | 0.000 | 0.000 | 0.000 | 0.050 | 0.024 | 0.007 | 0.008 | 0.000 |
| **f__Aeromonadaceae** | 0.000 | 0.000 | 0.000 | 0.000 | 0.000 | 0.000 | 0.000 | 0.000 | 0.000 | 0.013 | 0.124 | 0.000 |
| **g__Oribacterium** | 0.000 | 0.000 | 0.000 | 0.000 | 0.000 | 0.127 | 0.000 | 0.000 | 0.000 | 0.000 | 0.008 | 0.000 |
| **g__Actinobacillus** | 0.010 | 0.000 | 0.000 | 0.000 | 0.000 | 0.032 | 0.000 | 0.000 | 0.043 | 0.000 | 0.050 | 0.000 |
| **f__F16** | 0.048 | 0.071 | 0.000 | 0.000 | 0.014 | 0.000 | 0.000 | 0.000 | 0.000 | 0.000 | 0.000 | 0.000 |
| **o__Pseudomonadales** | 0.010 | 0.010 | 0.000 | 0.000 | 0.000 | 0.005 | 0.004 | 0.004 | 0.062 | 0.027 | 0.008 | 0.003 |
| **g__Ochrobactrum** | 0.000 | 0.000 | 0.000 | 0.000 | 0.000 | 0.090 | 0.000 | 0.021 | 0.014 | 0.007 | 0.000 | 0.000 |
| **c__Spirochaetes** | 0.000 | 0.000 | 0.000 | 0.000 | 0.000 | 0.037 | 0.083 | 0.004 | 0.000 | 0.007 | 0.000 | 0.000 |
| **g__Pseudoxanthomonas** | 0.000 | 0.000 | 0.000 | 0.000 | 0.014 | 0.026 | 0.000 | 0.050 | 0.029 | 0.007 | 0.000 | 0.003 |
| **g__[Ruminococcus]** | 0.029 | 0.092 | 0.000 | 0.000 | 0.000 | 0.000 | 0.000 | 0.000 | 0.000 | 0.007 | 0.000 | 0.000 |
| **g__Lewinella** | 0.000 | 0.000 | 0.000 | 0.000 | 0.000 | 0.000 | 0.000 | 0.000 | 0.000 | 0.013 | 0.107 | 0.007 |
| **g__Porphyromonas** | 0.029 | 0.082 | 0.000 | 0.000 | 0.000 | 0.000 | 0.000 | 0.004 | 0.005 | 0.000 | 0.008 | 0.000 |
| **g__Bordetella** | 0.068 | 0.020 | 0.000 | 0.000 | 0.000 | 0.000 | 0.000 | 0.004 | 0.000 | 0.000 | 0.000 | 0.033 |
| **f__PAUC26f** | 0.000 | 0.000 | 0.005 | 0.000 | 0.000 | 0.000 | 0.000 | 0.000 | 0.105 | 0.013 | 0.000 | 0.000 |
| **g__Phaeobacter** | 0.000 | 0.000 | 0.000 | 0.000 | 0.000 | 0.000 | 0.000 | 0.000 | 0.019 | 0.013 | 0.091 | 0.000 |
| **f__Caldilineaceae** | 0.000 | 0.000 | 0.000 | 0.000 | 0.014 | 0.000 | 0.000 | 0.032 | 0.014 | 0.040 | 0.017 | 0.000 |
| **o__JG30-KF-CM45** | 0.097 | 0.000 | 0.000 | 0.000 | 0.000 | 0.000 | 0.000 | 0.000 | 0.019 | 0.000 | 0.000 | 0.000 |
| **g__Shewanella** | 0.000 | 0.000 | 0.000 | 0.000 | 0.000 | 0.000 | 0.000 | 0.000 | 0.000 | 0.000 | 0.115 | 0.000 |
| **g__Myxococcus** | 0.019 | 0.000 | 0.000 | 0.000 | 0.000 | 0.000 | 0.000 | 0.000 | 0.000 | 0.094 | 0.000 | 0.000 |
| **g__Cloacibacterium** | 0.000 | 0.000 | 0.000 | 0.012 | 0.000 | 0.016 | 0.000 | 0.000 | 0.000 | 0.060 | 0.025 | 0.000 |
| **g__Rikenella** | 0.019 | 0.092 | 0.000 | 0.000 | 0.000 | 0.000 | 0.000 | 0.000 | 0.000 | 0.000 | 0.000 | 0.000 |
| **f__[Chromatiaceae]** | 0.000 | 0.000 | 0.000 | 0.000 | 0.000 | 0.000 | 0.109 | 0.000 | 0.000 | 0.000 | 0.000 | 0.000 |
| **g__Actinomycetospora** | 0.029 | 0.000 | 0.000 | 0.000 | 0.000 | 0.000 | 0.000 | 0.053 | 0.000 | 0.027 | 0.000 | 0.000 |
| **g__Lysobacter** | 0.010 | 0.000 | 0.000 | 0.000 | 0.000 | 0.000 | 0.000 | 0.050 | 0.014 | 0.027 | 0.008 | 0.000 |
| **o__Cytophagales** | 0.010 | 0.051 | 0.000 | 0.000 | 0.000 | 0.000 | 0.000 | 0.004 | 0.005 | 0.013 | 0.025 | 0.000 |
| **f__Synechococcaceae** | 0.000 | 0.051 | 0.000 | 0.012 | 0.028 | 0.000 | 0.000 | 0.000 | 0.014 | 0.000 | 0.000 | 0.000 |
| **f__Saprospiraceae** | 0.000 | 0.000 | 0.000 | 0.000 | 0.000 | 0.000 | 0.000 | 0.000 | 0.000 | 0.034 | 0.058 | 0.013 |
| **f__mitochondria** | 0.000 | 0.010 | 0.005 | 0.000 | 0.028 | 0.000 | 0.035 | 0.018 | 0.000 | 0.000 | 0.008 | 0.000 |
| **f__Desulfovibrionaceae** | 0.019 | 0.051 | 0.000 | 0.000 | 0.000 | 0.011 | 0.009 | 0.000 | 0.014 | 0.000 | 0.000 | 0.000 |
| **f__Nitrospiraceae** | 0.000 | 0.000 | 0.000 | 0.000 | 0.000 | 0.000 | 0.000 | 0.000 | 0.010 | 0.094 | 0.000 | 0.000 |
| **g__Nitrobacteria** | 0.000 | 0.000 | 0.000 | 0.000 | 0.000 | 0.074 | 0.000 | 0.000 | 0.000 | 0.000 | 0.000 | 0.030 |
| **g__Millisia** | 0.000 | 0.000 | 0.000 | 0.000 | 0.000 | 0.000 | 0.000 | 0.000 | 0.000 | 0.101 | 0.000 | 0.000 |
| **g__Bulleidia** | 0.000 | 0.000 | 0.000 | 0.000 | 0.000 | 0.000 | 0.000 | 0.099 | 0.000 | 0.000 | 0.000 | 0.000 |
| **g__Wautersiella** | 0.000 | 0.000 | 0.000 | 0.024 | 0.014 | 0.000 | 0.000 | 0.060 | 0.000 | 0.000 | 0.000 | 0.000 |
| **g__Selenomonas** | 0.010 | 0.031 | 0.000 | 0.000 | 0.000 | 0.037 | 0.000 | 0.000 | 0.000 | 0.020 | 0.000 | 0.000 |
| **g__Dermacoccus** | 0.019 | 0.000 | 0.000 | 0.000 | 0.000 | 0.000 | 0.000 | 0.035 | 0.010 | 0.007 | 0.017 | 0.010 |
| **f__OM60** | 0.000 | 0.041 | 0.032 | 0.000 | 0.000 | 0.000 | 0.000 | 0.000 | 0.000 | 0.007 | 0.017 | 0.000 |
| **g__Peredibacter** | 0.000 | 0.000 | 0.027 | 0.047 | 0.014 | 0.000 | 0.000 | 0.000 | 0.000 | 0.000 | 0.008 | 0.000 |
| **f__Pseudanabaenaceae** | 0.000 | 0.000 | 0.000 | 0.000 | 0.014 | 0.063 | 0.000 | 0.000 | 0.000 | 0.000 | 0.017 | 0.000 |
| **g__Leucobacter** | 0.000 | 0.000 | 0.000 | 0.000 | 0.000 | 0.000 | 0.083 | 0.011 | 0.000 | 0.000 | 0.000 | 0.000 |
| **g__Flavobacterium** | 0.010 | 0.000 | 0.000 | 0.000 | 0.000 | 0.053 | 0.000 | 0.018 | 0.000 | 0.007 | 0.000 | 0.007 |
| **g__Methyloversatilis** | 0.000 | 0.020 | 0.000 | 0.000 | 0.000 | 0.005 | 0.004 | 0.039 | 0.024 | 0.000 | 0.000 | 0.000 |
| **g__Paenibacillus** | 0.000 | 0.092 | 0.000 | 0.000 | 0.000 | 0.000 | 0.000 | 0.000 | 0.000 | 0.000 | 0.000 | 0.000 |
| **f__Deinococcaceae** | 0.010 | 0.000 | 0.000 | 0.000 | 0.000 | 0.000 | 0.000 | 0.014 | 0.067 | 0.000 | 0.000 | 0.000 |
| **f__Leptotrichiaceae** | 0.000 | 0.010 | 0.000 | 0.000 | 0.000 | 0.079 | 0.000 | 0.000 | 0.000 | 0.000 | 0.000 | 0.000 |
| **g__Aggregatibacter** | 0.000 | 0.010 | 0.000 | 0.000 | 0.000 | 0.079 | 0.000 | 0.000 | 0.000 | 0.000 | 0.000 | 0.000 |
| **g__Rubrobacter** | 0.000 | 0.000 | 0.005 | 0.000 | 0.000 | 0.000 | 0.000 | 0.050 | 0.033 | 0.000 | 0.000 | 0.000 |
| **g__Dehalobacterium** | 0.000 | 0.061 | 0.000 | 0.000 | 0.000 | 0.000 | 0.000 | 0.000 | 0.000 | 0.027 | 0.000 | 0.000 |
| **f__Flammeovirgaceae** | 0.000 | 0.010 | 0.000 | 0.012 | 0.000 | 0.000 | 0.000 | 0.000 | 0.010 | 0.020 | 0.033 | 0.000 |
| **f__OCS155** | 0.029 | 0.010 | 0.005 | 0.012 | 0.028 | 0.000 | 0.000 | 0.000 | 0.000 | 0.000 | 0.000 | 0.000 |
| **f__Xanthobacteraceae** | 0.000 | 0.000 | 0.000 | 0.000 | 0.000 | 0.069 | 0.000 | 0.000 | 0.000 | 0.007 | 0.000 | 0.007 |
| **g__Oceanospirillum** | 0.000 | 0.000 | 0.032 | 0.000 | 0.042 | 0.000 | 0.000 | 0.000 | 0.000 | 0.000 | 0.000 | 0.007 |
| **g__HTCC** | 0.000 | 0.031 | 0.005 | 0.012 | 0.000 | 0.000 | 0.000 | 0.000 | 0.019 | 0.013 | 0.000 | 0.000 |
| **f__[Paraprevotellaceae]** | 0.019 | 0.000 | 0.000 | 0.000 | 0.000 | 0.021 | 0.026 | 0.000 | 0.000 | 0.013 | 0.000 | 0.000 |
| **g__Bermanella** | 0.000 | 0.000 | 0.000 | 0.024 | 0.042 | 0.000 | 0.000 | 0.000 | 0.000 | 0.013 | 0.000 | 0.000 |
| **c__Gemm-4** | 0.068 | 0.000 | 0.011 | 0.000 | 0.000 | 0.000 | 0.000 | 0.000 | 0.000 | 0.000 | 0.000 | 0.000 |
| **g__Nesterenkonia** | 0.048 | 0.000 | 0.000 | 0.000 | 0.000 | 0.000 | 0.000 | 0.000 | 0.010 | 0.020 | 0.000 | 0.000 |
| **f__OM27** | 0.000 | 0.000 | 0.000 | 0.000 | 0.000 | 0.000 | 0.000 | 0.000 | 0.000 | 0.007 | 0.058 | 0.013 |
| **f__Pirellulaceae** | 0.010 | 0.010 | 0.005 | 0.000 | 0.028 | 0.000 | 0.000 | 0.000 | 0.000 | 0.007 | 0.000 | 0.016 |
| **g__Erythromicrobium** | 0.000 | 0.000 | 0.000 | 0.000 | 0.000 | 0.000 | 0.000 | 0.000 | 0.048 | 0.020 | 0.008 | 0.000 |
| **f__Mycoplasmataceae** | 0.000 | 0.000 | 0.000 | 0.000 | 0.028 | 0.047 | 0.000 | 0.000 | 0.000 | 0.000 | 0.000 | 0.000 |
| **g__Clostridium** | 0.000 | 0.000 | 0.000 | 0.000 | 0.000 | 0.000 | 0.061 | 0.007 | 0.000 | 0.007 | 0.000 | 0.000 |
| **g__Gordonia** | 0.000 | 0.000 | 0.000 | 0.000 | 0.000 | 0.000 | 0.000 | 0.018 | 0.057 | 0.000 | 0.000 | 0.000 |
| **g__Streptomyces** | 0.000 | 0.000 | 0.000 | 0.000 | 0.000 | 0.000 | 0.000 | 0.046 | 0.029 | 0.000 | 0.000 | 0.000 |
| **g__Anaerovorax** | 0.019 | 0.031 | 0.000 | 0.000 | 0.000 | 0.000 | 0.000 | 0.000 | 0.000 | 0.000 | 0.025 | 0.000 |
| **g__Marinobacter** | 0.000 | 0.010 | 0.000 | 0.012 | 0.028 | 0.000 | 0.000 | 0.000 | 0.000 | 0.000 | 0.025 | 0.000 |
| **g__Maricaulis** | 0.000 | 0.000 | 0.000 | 0.000 | 0.000 | 0.000 | 0.000 | 0.000 | 0.000 | 0.000 | 0.074 | 0.000 |
| **f__Hyphomicrobiaceae** | 0.000 | 0.000 | 0.011 | 0.000 | 0.000 | 0.000 | 0.000 | 0.011 | 0.005 | 0.013 | 0.033 | 0.000 |
| **g__Colwellia** | 0.000 | 0.000 | 0.000 | 0.035 | 0.028 | 0.000 | 0.000 | 0.000 | 0.000 | 0.000 | 0.008 | 0.000 |
| **g__Dietzia** | 0.000 | 0.000 | 0.000 | 0.000 | 0.000 | 0.000 | 0.000 | 0.071 | 0.000 | 0.000 | 0.000 | 0.000 |
| **o__Acidimicrobiales** | 0.000 | 0.000 | 0.011 | 0.000 | 0.000 | 0.000 | 0.031 | 0.000 | 0.014 | 0.007 | 0.008 | 0.000 |
| **g__Xanthomonas** | 0.000 | 0.000 | 0.000 | 0.000 | 0.000 | 0.000 | 0.000 | 0.018 | 0.000 | 0.000 | 0.050 | 0.000 |
| **g__Nocardioides** | 0.010 | 0.000 | 0.000 | 0.000 | 0.000 | 0.000 | 0.000 | 0.057 | 0.000 | 0.000 | 0.000 | 0.000 |
| **f__Rhodothermaceae** | 0.000 | 0.000 | 0.000 | 0.000 | 0.000 | 0.000 | 0.000 | 0.021 | 0.005 | 0.040 | 0.000 | 0.000 |
| **o__Sva0725** | 0.000 | 0.000 | 0.000 | 0.012 | 0.000 | 0.000 | 0.000 | 0.000 | 0.048 | 0.007 | 0.000 | 0.000 |
| **g__Haliea** | 0.000 | 0.041 | 0.000 | 0.000 | 0.000 | 0.000 | 0.000 | 0.000 | 0.000 | 0.000 | 0.025 | 0.000 |
| **f__Campylobacteraceae** | 0.010 | 0.000 | 0.016 | 0.012 | 0.028 | 0.000 | 0.000 | 0.000 | 0.000 | 0.000 | 0.000 | 0.000 |
| **f__Clostridiaceae** | 0.010 | 0.000 | 0.000 | 0.012 | 0.014 | 0.000 | 0.000 | 0.000 | 0.010 | 0.020 | 0.000 | 0.000 |
| **f__Bacteroidaceae** | 0.010 | 0.000 | 0.000 | 0.000 | 0.000 | 0.016 | 0.009 | 0.011 | 0.000 | 0.020 | 0.000 | 0.000 |
| **o__Chroococcales** | 0.000 | 0.031 | 0.000 | 0.000 | 0.000 | 0.000 | 0.004 | 0.000 | 0.010 | 0.020 | 0.000 | 0.000 |
| **f__Brucellaceae** | 0.000 | 0.000 | 0.000 | 0.000 | 0.000 | 0.005 | 0.000 | 0.035 | 0.024 | 0.000 | 0.000 | 0.000 |
| **g__Ruegeria** | 0.000 | 0.000 | 0.000 | 0.000 | 0.000 | 0.000 | 0.000 | 0.000 | 0.000 | 0.007 | 0.058 | 0.000 |
| **f__Fusobacteriaceae** | 0.000 | 0.000 | 0.000 | 0.000 | 0.000 | 0.026 | 0.000 | 0.028 | 0.000 | 0.007 | 0.000 | 0.000 |
| **g__Fibrobacter** | 0.000 | 0.031 | 0.005 | 0.000 | 0.000 | 0.021 | 0.000 | 0.004 | 0.000 | 0.000 | 0.000 | 0.000 |
| **g__Ferrimonas** | 0.000 | 0.000 | 0.048 | 0.012 | 0.000 | 0.000 | 0.000 | 0.000 | 0.000 | 0.000 | 0.000 | 0.000 |
| **f__Piscirickettsiaceae** | 0.019 | 0.020 | 0.000 | 0.000 | 0.000 | 0.000 | 0.000 | 0.000 | 0.019 | 0.000 | 0.000 | 0.000 |
| **f__Aerococcaceae** | 0.010 | 0.010 | 0.000 | 0.000 | 0.000 | 0.005 | 0.000 | 0.007 | 0.000 | 0.000 | 0.025 | 0.000 |
| **g__Knoellia** | 0.010 | 0.000 | 0.000 | 0.000 | 0.000 | 0.000 | 0.000 | 0.000 | 0.000 | 0.047 | 0.000 | 0.000 |
| **o__Desulfovibrionales** | 0.010 | 0.020 | 0.000 | 0.000 | 0.000 | 0.011 | 0.004 | 0.000 | 0.005 | 0.007 | 0.000 | 0.000 |
| **c__Chloroplast** | 0.000 | 0.000 | 0.032 | 0.000 | 0.000 | 0.000 | 0.004 | 0.000 | 0.019 | 0.000 | 0.000 | 0.000 |
| **o__Myxococcales** | 0.010 | 0.000 | 0.000 | 0.000 | 0.000 | 0.000 | 0.000 | 0.025 | 0.000 | 0.020 | 0.000 | 0.000 |
| **g__Olsenella** | 0.010 | 0.010 | 0.000 | 0.000 | 0.000 | 0.005 | 0.004 | 0.000 | 0.000 | 0.000 | 0.025 | 0.000 |
| **o__MIZ46** | 0.010 | 0.020 | 0.000 | 0.000 | 0.000 | 0.011 | 0.000 | 0.000 | 0.000 | 0.013 | 0.000 | 0.000 |
| **g__Trueperella** | 0.000 | 0.000 | 0.000 | 0.000 | 0.000 | 0.053 | 0.000 | 0.000 | 0.000 | 0.000 | 0.000 | 0.000 |
| **g__Comamonas** | 0.000 | 0.000 | 0.000 | 0.000 | 0.000 | 0.021 | 0.031 | 0.000 | 0.000 | 0.000 | 0.000 | 0.000 |
| **c__Ellin6529** | 0.000 | 0.000 | 0.000 | 0.000 | 0.000 | 0.000 | 0.000 | 0.000 | 0.000 | 0.027 | 0.025 | 0.000 |
| **g__Spongiibacter** | 0.000 | 0.000 | 0.000 | 0.024 | 0.028 | 0.000 | 0.000 | 0.000 | 0.000 | 0.000 | 0.000 | 0.000 |
| **g__Providencia** | 0.000 | 0.051 | 0.000 | 0.000 | 0.000 | 0.000 | 0.000 | 0.000 | 0.000 | 0.000 | 0.000 | 0.000 |
| **g__Cohaesibacter** | 0.000 | 0.000 | 0.043 | 0.000 | 0.000 | 0.000 | 0.000 | 0.000 | 0.000 | 0.007 | 0.000 | 0.000 |
| **g__Oceanicaulis** | 0.000 | 0.000 | 0.000 | 0.000 | 0.000 | 0.000 | 0.000 | 0.000 | 0.000 | 0.013 | 0.017 | 0.020 |
| **g__Pseudonocardia** | 0.000 | 0.000 | 0.000 | 0.000 | 0.000 | 0.000 | 0.000 | 0.000 | 0.000 | 0.000 | 0.050 | 0.000 |
| **c__ABY1** | 0.000 | 0.000 | 0.000 | 0.000 | 0.000 | 0.000 | 0.000 | 0.000 | 0.000 | 0.000 | 0.050 | 0.000 |
| **g__Lautropia** | 0.029 | 0.010 | 0.000 | 0.000 | 0.000 | 0.000 | 0.000 | 0.000 | 0.010 | 0.000 | 0.000 | 0.000 |
| **c__Gitt-GS-136** | 0.048 | 0.000 | 0.000 | 0.000 | 0.000 | 0.000 | 0.000 | 0.000 | 0.000 | 0.000 | 0.000 | 0.000 |
| **o__Rhodobacterales** | 0.000 | 0.000 | 0.000 | 0.000 | 0.000 | 0.000 | 0.000 | 0.000 | 0.005 | 0.007 | 0.033 | 0.003 |
| **g__Idiomarina** | 0.000 | 0.000 | 0.005 | 0.012 | 0.014 | 0.000 | 0.000 | 0.000 | 0.000 | 0.000 | 0.017 | 0.000 |
| **g__Mogibacterium** | 0.000 | 0.000 | 0.000 | 0.000 | 0.014 | 0.000 | 0.000 | 0.000 | 0.000 | 0.034 | 0.000 | 0.000 |
| **g__Erysipelothrix** | 0.000 | 0.000 | 0.011 | 0.000 | 0.000 | 0.000 | 0.000 | 0.000 | 0.005 | 0.007 | 0.025 | 0.000 |
| **g__Desulfarculus** | 0.010 | 0.000 | 0.000 | 0.000 | 0.000 | 0.000 | 0.000 | 0.004 | 0.000 | 0.034 | 0.000 | 0.000 |
| **g__Facklamia** | 0.019 | 0.000 | 0.000 | 0.000 | 0.000 | 0.000 | 0.000 | 0.018 | 0.000 | 0.007 | 0.000 | 0.000 |
| **g__Achromobacter** | 0.000 | 0.000 | 0.000 | 0.000 | 0.000 | 0.000 | 0.018 | 0.000 | 0.000 | 0.000 | 0.000 | 0.026 |
| **f__Desulfobulbaceae** | 0.010 | 0.000 | 0.000 | 0.000 | 0.000 | 0.000 | 0.000 | 0.000 | 0.033 | 0.000 | 0.000 | 0.000 |
| **o__Chlamydiales** | 0.000 | 0.000 | 0.000 | 0.000 | 0.000 | 0.042 | 0.000 | 0.000 | 0.000 | 0.000 | 0.000 | 0.000 |
| **g__Pseudochrobactrum** | 0.000 | 0.000 | 0.000 | 0.000 | 0.000 | 0.042 | 0.000 | 0.000 | 0.000 | 0.000 | 0.000 | 0.000 |
| **g__Alcanivorax** | 0.000 | 0.000 | 0.000 | 0.012 | 0.014 | 0.000 | 0.000 | 0.000 | 0.000 | 0.000 | 0.017 | 0.000 |
| **g__Krokinobacter** | 0.000 | 0.000 | 0.000 | 0.000 | 0.000 | 0.000 | 0.000 | 0.000 | 0.000 | 0.000 | 0.041 | 0.000 |
| **g__Solibacillus** | 0.000 | 0.000 | 0.000 | 0.000 | 0.000 | 0.000 | 0.000 | 0.000 | 0.000 | 0.000 | 0.041 | 0.000 |
| **g__Pseudoruegeria** | 0.000 | 0.000 | 0.000 | 0.000 | 0.000 | 0.000 | 0.000 | 0.000 | 0.000 | 0.000 | 0.041 | 0.000 |
| **g__Tepidimonas** | 0.000 | 0.000 | 0.000 | 0.000 | 0.000 | 0.000 | 0.000 | 0.000 | 0.000 | 0.000 | 0.041 | 0.000 |
| **f__Actinomycetaceae** | 0.000 | 0.000 | 0.011 | 0.000 | 0.000 | 0.016 | 0.000 | 0.000 | 0.014 | 0.000 | 0.000 | 0.000 |
| **g__Anaeroplasma** | 0.000 | 0.041 | 0.000 | 0.000 | 0.000 | 0.000 | 0.000 | 0.000 | 0.000 | 0.000 | 0.000 | 0.000 |
| **g__Adlercreutzia** | 0.019 | 0.020 | 0.000 | 0.000 | 0.000 | 0.000 | 0.000 | 0.000 | 0.000 | 0.000 | 0.000 | 0.000 |
| **g__Epulopiscium** | 0.000 | 0.000 | 0.000 | 0.000 | 0.000 | 0.000 | 0.039 | 0.000 | 0.000 | 0.000 | 0.000 | 0.000 |
| **f__Halomonadaceae** | 0.000 | 0.010 | 0.005 | 0.000 | 0.000 | 0.000 | 0.000 | 0.014 | 0.010 | 0.000 | 0.000 | 0.000 |
| **g__Gardnerella** | 0.000 | 0.000 | 0.000 | 0.000 | 0.000 | 0.000 | 0.000 | 0.039 | 0.000 | 0.000 | 0.000 | 0.000 |
| **g__Methylotenera** | 0.000 | 0.000 | 0.000 | 0.000 | 0.000 | 0.000 | 0.000 | 0.039 | 0.000 | 0.000 | 0.000 | 0.000 |
| **g__Myroides** | 0.000 | 0.000 | 0.000 | 0.000 | 0.014 | 0.000 | 0.000 | 0.000 | 0.000 | 0.000 | 0.025 | 0.000 |
| **f__Coriobacteriaceae** | 0.010 | 0.000 | 0.000 | 0.000 | 0.000 | 0.005 | 0.004 | 0.000 | 0.019 | 0.000 | 0.000 | 0.000 |
| **g__Salegentibacter** | 0.000 | 0.000 | 0.000 | 0.024 | 0.014 | 0.000 | 0.000 | 0.000 | 0.000 | 0.000 | 0.000 | 0.000 |
| **f__Sinobacteraceae** | 0.000 | 0.000 | 0.000 | 0.000 | 0.014 | 0.000 | 0.000 | 0.007 | 0.000 | 0.000 | 0.017 | 0.000 |
| **g__Nitrosopumilus** | 0.000 | 0.000 | 0.000 | 0.000 | 0.000 | 0.000 | 0.000 | 0.004 | 0.033 | 0.000 | 0.000 | 0.000 |
| **g__Aeromonas** | 0.000 | 0.000 | 0.000 | 0.012 | 0.000 | 0.000 | 0.000 | 0.000 | 0.005 | 0.020 | 0.000 | 0.000 |
| **o__MLE1-12** | 0.000 | 0.000 | 0.000 | 0.012 | 0.000 | 0.000 | 0.000 | 0.000 | 0.000 | 0.000 | 0.025 | 0.000 |
| **g__Phenylobacterium** | 0.000 | 0.000 | 0.005 | 0.024 | 0.000 | 0.000 | 0.000 | 0.000 | 0.000 | 0.007 | 0.000 | 0.000 |
| **g__Defluviicoccus** | 0.000 | 0.000 | 0.000 | 0.000 | 0.000 | 0.000 | 0.000 | 0.035 | 0.000 | 0.000 | 0.000 | 0.000 |
| **c__Actinobacteria** | 0.000 | 0.000 | 0.000 | 0.000 | 0.000 | 0.000 | 0.000 | 0.018 | 0.010 | 0.007 | 0.000 | 0.000 |
| **g__Marinibacillus** | 0.000 | 0.000 | 0.005 | 0.000 | 0.000 | 0.000 | 0.000 | 0.028 | 0.000 | 0.000 | 0.000 | 0.000 |
| **g__Pontibacter** | 0.000 | 0.000 | 0.000 | 0.000 | 0.000 | 0.000 | 0.000 | 0.000 | 0.000 | 0.034 | 0.000 | 0.000 |
| **g__Mycoplana** | 0.000 | 0.000 | 0.000 | 0.000 | 0.000 | 0.000 | 0.000 | 0.000 | 0.000 | 0.007 | 0.017 | 0.010 |
| **g__Rickettsiella** | 0.000 | 0.000 | 0.000 | 0.000 | 0.000 | 0.000 | 0.000 | 0.032 | 0.000 | 0.000 | 0.000 | 0.000 |
| **g__Hyphomicrobium** | 0.000 | 0.000 | 0.000 | 0.000 | 0.000 | 0.000 | 0.000 | 0.025 | 0.000 | 0.007 | 0.000 | 0.000 |
| **g__Mesoflavibacter** | 0.000 | 0.000 | 0.000 | 0.000 | 0.000 | 0.000 | 0.000 | 0.000 | 0.000 | 0.007 | 0.025 | 0.000 |
| **f__Chitinophagaceae** | 0.000 | 0.000 | 0.000 | 0.000 | 0.000 | 0.000 | 0.004 | 0.000 | 0.000 | 0.027 | 0.000 | 0.000 |
| **f__Cenarchaeaceae** | 0.000 | 0.000 | 0.000 | 0.012 | 0.000 | 0.000 | 0.000 | 0.014 | 0.005 | 0.000 | 0.000 | 0.000 |
| **g__Peptostreptococcus** | 0.000 | 0.031 | 0.000 | 0.000 | 0.000 | 0.000 | 0.000 | 0.000 | 0.000 | 0.000 | 0.000 | 0.000 |
| **g__Arenimonas** | 0.000 | 0.031 | 0.000 | 0.000 | 0.000 | 0.000 | 0.000 | 0.000 | 0.000 | 0.000 | 0.000 | 0.000 |
| **f__Pseudonocardiaceae** | 0.010 | 0.010 | 0.000 | 0.000 | 0.000 | 0.000 | 0.000 | 0.011 | 0.000 | 0.000 | 0.000 | 0.000 |
| **g__Salisaeta** | 0.000 | 0.010 | 0.000 | 0.000 | 0.000 | 0.000 | 0.000 | 0.000 | 0.000 | 0.020 | 0.000 | 0.000 |
| **g__Thalassospira** | 0.000 | 0.000 | 0.016 | 0.000 | 0.014 | 0.000 | 0.000 | 0.000 | 0.000 | 0.000 | 0.000 | 0.000 |
| **g__02d06** | 0.000 | 0.000 | 0.000 | 0.000 | 0.000 | 0.005 | 0.000 | 0.000 | 0.000 | 0.000 | 0.025 | 0.000 |
| **f__Polyangiaceae** | 0.000 | 0.000 | 0.005 | 0.000 | 0.000 | 0.000 | 0.000 | 0.018 | 0.000 | 0.007 | 0.000 | 0.000 |
| **g__Granulicatella** | 0.019 | 0.010 | 0.000 | 0.000 | 0.000 | 0.000 | 0.000 | 0.000 | 0.000 | 0.000 | 0.000 | 0.000 |
| **g__Inquilinus** | 0.019 | 0.010 | 0.000 | 0.000 | 0.000 | 0.000 | 0.000 | 0.000 | 0.000 | 0.000 | 0.000 | 0.000 |
| **o__Solirubrobacterales** | 0.010 | 0.000 | 0.000 | 0.000 | 0.000 | 0.005 | 0.000 | 0.014 | 0.000 | 0.000 | 0.000 | 0.000 |
| **g__p-75-a5** | 0.029 | 0.000 | 0.000 | 0.000 | 0.000 | 0.000 | 0.000 | 0.000 | 0.000 | 0.000 | 0.000 | 0.000 |
| **g__Petrimonas** | 0.000 | 0.000 | 0.000 | 0.000 | 0.014 | 0.000 | 0.000 | 0.000 | 0.000 | 0.007 | 0.008 | 0.000 |
| **g__Candidatus_Phlomobacter** | 0.000 | 0.000 | 0.000 | 0.000 | 0.000 | 0.000 | 0.000 | 0.000 | 0.029 | 0.000 | 0.000 | 0.000 |
| **g__Rubritalea** | 0.000 | 0.000 | 0.000 | 0.000 | 0.000 | 0.000 | 0.000 | 0.000 | 0.029 | 0.000 | 0.000 | 0.000 |
| **g__Megasphaera** | 0.000 | 0.020 | 0.000 | 0.000 | 0.000 | 0.000 | 0.000 | 0.000 | 0.000 | 0.000 | 0.008 | 0.000 |
| **f__[Marinicellaceae]** | 0.000 | 0.020 | 0.000 | 0.000 | 0.000 | 0.000 | 0.000 | 0.000 | 0.000 | 0.000 | 0.008 | 0.000 |
| **f__Propionibacteriaceae** | 0.010 | 0.000 | 0.000 | 0.000 | 0.000 | 0.011 | 0.000 | 0.000 | 0.000 | 0.000 | 0.008 | 0.000 |
| **f__Marinilabiaceae** | 0.000 | 0.000 | 0.000 | 0.000 | 0.014 | 0.000 | 0.000 | 0.014 | 0.000 | 0.000 | 0.000 | 0.000 |
| **p__Acidobacteria** | 0.019 | 0.000 | 0.000 | 0.000 | 0.000 | 0.000 | 0.009 | 0.000 | 0.000 | 0.000 | 0.000 | 0.000 |
| **g__Schlegelella** | 0.000 | 0.000 | 0.000 | 0.000 | 0.028 | 0.000 | 0.000 | 0.000 | 0.000 | 0.000 | 0.000 | 0.000 |
| **g__Psychrobacter** | 0.000 | 0.000 | 0.000 | 0.000 | 0.028 | 0.000 | 0.000 | 0.000 | 0.000 | 0.000 | 0.000 | 0.000 |
| **f__JdFBGBact** | 0.000 | 0.000 | 0.000 | 0.000 | 0.000 | 0.000 | 0.000 | 0.000 | 0.014 | 0.013 | 0.000 | 0.000 |
| **c__Anaerolineae** | 0.000 | 0.010 | 0.000 | 0.000 | 0.000 | 0.000 | 0.000 | 0.011 | 0.000 | 0.007 | 0.000 | 0.000 |
| **o__Chlorophyta** | 0.000 | 0.000 | 0.000 | 0.000 | 0.000 | 0.000 | 0.000 | 0.000 | 0.000 | 0.027 | 0.000 | 0.000 |
| **g__Reyranella** | 0.000 | 0.000 | 0.000 | 0.000 | 0.000 | 0.000 | 0.000 | 0.000 | 0.000 | 0.027 | 0.000 | 0.000 |
| **o__Spirobacillales** | 0.000 | 0.000 | 0.000 | 0.000 | 0.000 | 0.000 | 0.000 | 0.000 | 0.000 | 0.027 | 0.000 | 0.000 |
| **g__Candidatus_Rhabdochlamydia** | 0.000 | 0.000 | 0.000 | 0.000 | 0.000 | 0.026 | 0.000 | 0.000 | 0.000 | 0.000 | 0.000 | 0.000 |
| **g__Paenisporosarcina** | 0.000 | 0.000 | 0.000 | 0.000 | 0.000 | 0.026 | 0.000 | 0.000 | 0.000 | 0.000 | 0.000 | 0.000 |
| **g__Candidatus_Arthromitus** | 0.000 | 0.000 | 0.000 | 0.000 | 0.000 | 0.026 | 0.000 | 0.000 | 0.000 | 0.000 | 0.000 | 0.000 |
| **g__Aquabacterium** | 0.000 | 0.000 | 0.000 | 0.000 | 0.000 | 0.000 | 0.000 | 0.007 | 0.019 | 0.000 | 0.000 | 0.000 |
| **g__Kingella** | 0.000 | 0.000 | 0.000 | 0.000 | 0.000 | 0.000 | 0.004 | 0.021 | 0.000 | 0.000 | 0.000 | 0.000 |
| **g__Anaerospora** | 0.000 | 0.000 | 0.000 | 0.012 | 0.014 | 0.000 | 0.000 | 0.000 | 0.000 | 0.000 | 0.000 | 0.000 |
| **f__Coxiellaceae** | 0.000 | 0.020 | 0.000 | 0.000 | 0.000 | 0.000 | 0.000 | 0.000 | 0.005 | 0.000 | 0.000 | 0.000 |
| **f__0319-6G20** | 0.000 | 0.000 | 0.000 | 0.000 | 0.000 | 0.000 | 0.000 | 0.025 | 0.000 | 0.000 | 0.000 | 0.000 |
| **o__BD7-3** | 0.000 | 0.000 | 0.000 | 0.000 | 0.000 | 0.000 | 0.000 | 0.000 | 0.000 | 0.000 | 0.025 | 0.000 |
| **f__Phyllobacteriaceae** | 0.000 | 0.000 | 0.000 | 0.000 | 0.000 | 0.000 | 0.000 | 0.000 | 0.000 | 0.000 | 0.025 | 0.000 |
| **g__Hoeflea** | 0.000 | 0.000 | 0.000 | 0.000 | 0.000 | 0.000 | 0.000 | 0.000 | 0.000 | 0.000 | 0.025 | 0.000 |
| **g__Oceanisphaera** | 0.000 | 0.000 | 0.000 | 0.000 | 0.000 | 0.000 | 0.000 | 0.000 | 0.000 | 0.000 | 0.025 | 0.000 |
| **g__Marinicella** | 0.000 | 0.000 | 0.000 | 0.000 | 0.000 | 0.000 | 0.000 | 0.000 | 0.000 | 0.000 | 0.025 | 0.000 |
| **o__AKIW781** | 0.019 | 0.000 | 0.000 | 0.000 | 0.000 | 0.000 | 0.000 | 0.000 | 0.005 | 0.000 | 0.000 | 0.000 |
| **o__Rhodophyta** | 0.000 | 0.000 | 0.000 | 0.000 | 0.000 | 0.000 | 0.000 | 0.000 | 0.024 | 0.000 | 0.000 | 0.000 |
| **g__Xenococcus** | 0.000 | 0.000 | 0.000 | 0.000 | 0.000 | 0.000 | 0.000 | 0.000 | 0.024 | 0.000 | 0.000 | 0.000 |
| **g__Eikenella** | 0.000 | 0.000 | 0.016 | 0.000 | 0.000 | 0.000 | 0.000 | 0.007 | 0.000 | 0.000 | 0.000 | 0.000 |
| **o__mle1-48** | 0.000 | 0.000 | 0.000 | 0.000 | 0.000 | 0.000 | 0.000 | 0.000 | 0.014 | 0.000 | 0.008 | 0.000 |
| **f__Methanobacteriaceae** | 0.000 | 0.000 | 0.000 | 0.000 | 0.000 | 0.000 | 0.022 | 0.000 | 0.000 | 0.000 | 0.000 | 0.000 |
| **o__GMD14H09** | 0.000 | 0.000 | 0.000 | 0.000 | 0.000 | 0.000 | 0.000 | 0.000 | 0.000 | 0.013 | 0.008 | 0.000 |
| **g__Devriesea** | 0.000 | 0.000 | 0.000 | 0.000 | 0.000 | 0.000 | 0.000 | 0.021 | 0.000 | 0.000 | 0.000 | 0.000 |
| **f__Micromonosporaceae** | 0.000 | 0.000 | 0.000 | 0.000 | 0.000 | 0.000 | 0.000 | 0.021 | 0.000 | 0.000 | 0.000 | 0.000 |
| **f__Geodermatophilaceae** | 0.000 | 0.000 | 0.000 | 0.000 | 0.000 | 0.021 | 0.000 | 0.000 | 0.000 | 0.000 | 0.000 | 0.000 |
| **g__Elizabethkingia** | 0.000 | 0.020 | 0.000 | 0.000 | 0.000 | 0.000 | 0.000 | 0.000 | 0.000 | 0.000 | 0.000 | 0.000 |
| **g__Tissierella_Soehngenia** | 0.000 | 0.000 | 0.000 | 0.000 | 0.000 | 0.000 | 0.000 | 0.000 | 0.000 | 0.020 | 0.000 | 0.000 |
| **f__Ellin6075** | 0.019 | 0.000 | 0.000 | 0.000 | 0.000 | 0.000 | 0.000 | 0.000 | 0.000 | 0.000 | 0.000 | 0.000 |
| **f__ntu14** | 0.019 | 0.000 | 0.000 | 0.000 | 0.000 | 0.000 | 0.000 | 0.000 | 0.000 | 0.000 | 0.000 | 0.000 |
| **g__Alicyclobacillus** | 0.019 | 0.000 | 0.000 | 0.000 | 0.000 | 0.000 | 0.000 | 0.000 | 0.000 | 0.000 | 0.000 | 0.000 |
| **g__ph2** | 0.019 | 0.000 | 0.000 | 0.000 | 0.000 | 0.000 | 0.000 | 0.000 | 0.000 | 0.000 | 0.000 | 0.000 |
| **g__Microbispora** | 0.000 | 0.000 | 0.000 | 0.000 | 0.000 | 0.000 | 0.000 | 0.000 | 0.019 | 0.000 | 0.000 | 0.000 |
| **g__Blastomonas** | 0.000 | 0.000 | 0.000 | 0.000 | 0.000 | 0.000 | 0.000 | 0.000 | 0.019 | 0.000 | 0.000 | 0.000 |
| **g__Hymenobacter** | 0.000 | 0.010 | 0.000 | 0.000 | 0.000 | 0.000 | 0.000 | 0.000 | 0.000 | 0.000 | 0.008 | 0.000 |
| **c__PAUC37f** | 0.000 | 0.000 | 0.000 | 0.000 | 0.000 | 0.000 | 0.000 | 0.018 | 0.000 | 0.000 | 0.000 | 0.000 |
| **g__Rickettsia** | 0.000 | 0.000 | 0.000 | 0.000 | 0.000 | 0.000 | 0.000 | 0.018 | 0.000 | 0.000 | 0.000 | 0.000 |
| **f__Endozoicimonaceae** | 0.000 | 0.010 | 0.000 | 0.000 | 0.000 | 0.000 | 0.000 | 0.000 | 0.000 | 0.000 | 0.000 | 0.007 |
| **f__Hyphomonadaceae** | 0.000 | 0.000 | 0.000 | 0.000 | 0.000 | 0.000 | 0.000 | 0.000 | 0.000 | 0.000 | 0.017 | 0.000 |
| **g__Henriciella** | 0.000 | 0.000 | 0.000 | 0.000 | 0.000 | 0.000 | 0.000 | 0.000 | 0.000 | 0.000 | 0.017 | 0.000 |
| **g__Hyphomonas** | 0.000 | 0.000 | 0.000 | 0.000 | 0.000 | 0.000 | 0.000 | 0.000 | 0.000 | 0.000 | 0.017 | 0.000 |
| **g__Sandaracinobacter** | 0.000 | 0.000 | 0.000 | 0.000 | 0.000 | 0.000 | 0.000 | 0.000 | 0.000 | 0.000 | 0.017 | 0.000 |
| **g__Cystobacter** | 0.000 | 0.000 | 0.000 | 0.000 | 0.000 | 0.000 | 0.000 | 0.000 | 0.000 | 0.000 | 0.017 | 0.000 |
| **g__Roseivivax** | 0.000 | 0.000 | 0.000 | 0.012 | 0.000 | 0.000 | 0.004 | 0.000 | 0.000 | 0.000 | 0.000 | 0.000 |
| **g__Papillibacter** | 0.000 | 0.000 | 0.005 | 0.000 | 0.000 | 0.000 | 0.000 | 0.011 | 0.000 | 0.000 | 0.000 | 0.000 |
| **f__[Tissierellaceae]** | 0.000 | 0.000 | 0.000 | 0.000 | 0.000 | 0.016 | 0.000 | 0.000 | 0.000 | 0.000 | 0.000 | 0.000 |
| **f__Halobacteriaceae** | 0.000 | 0.000 | 0.005 | 0.000 | 0.000 | 0.000 | 0.000 | 0.000 | 0.000 | 0.007 | 0.000 | 0.003 |
| **g__Alcaligenes** | 0.000 | 0.000 | 0.000 | 0.000 | 0.000 | 0.000 | 0.000 | 0.007 | 0.000 | 0.000 | 0.008 | 0.000 |
| **g__Rhodococcus** | 0.000 | 0.000 | 0.000 | 0.000 | 0.000 | 0.005 | 0.000 | 0.000 | 0.010 | 0.000 | 0.000 | 0.000 |
| **f__koll13** | 0.000 | 0.000 | 0.000 | 0.000 | 0.000 | 0.000 | 0.000 | 0.000 | 0.014 | 0.000 | 0.000 | 0.000 |
| **g__mixed** | 0.000 | 0.000 | 0.000 | 0.000 | 0.000 | 0.000 | 0.000 | 0.000 | 0.014 | 0.000 | 0.000 | 0.000 |
| **g__R18-435** | 0.000 | 0.000 | 0.000 | 0.000 | 0.000 | 0.000 | 0.000 | 0.000 | 0.014 | 0.000 | 0.000 | 0.000 |
| **g__Clostridium** | 0.000 | 0.000 | 0.000 | 0.000 | 0.000 | 0.000 | 0.000 | 0.014 | 0.000 | 0.000 | 0.000 | 0.000 |
| **f__Beijerinckiaceae** | 0.000 | 0.000 | 0.000 | 0.000 | 0.000 | 0.000 | 0.000 | 0.014 | 0.000 | 0.000 | 0.000 | 0.000 |
| **g__Afipia** | 0.000 | 0.000 | 0.000 | 0.000 | 0.000 | 0.000 | 0.000 | 0.014 | 0.000 | 0.000 | 0.000 | 0.000 |
| **g__Crenothrix** | 0.000 | 0.000 | 0.000 | 0.000 | 0.000 | 0.000 | 0.000 | 0.014 | 0.000 | 0.000 | 0.000 | 0.000 |
| **g__Sediminibacterium** | 0.000 | 0.000 | 0.000 | 0.000 | 0.014 | 0.000 | 0.000 | 0.000 | 0.000 | 0.000 | 0.000 | 0.000 |
| **f__A4b** | 0.000 | 0.000 | 0.000 | 0.000 | 0.014 | 0.000 | 0.000 | 0.000 | 0.000 | 0.000 | 0.000 | 0.000 |
| **o__CAB-I** | 0.000 | 0.000 | 0.000 | 0.000 | 0.014 | 0.000 | 0.000 | 0.000 | 0.000 | 0.000 | 0.000 | 0.000 |
| **g__Exiguobacterium** | 0.000 | 0.000 | 0.000 | 0.000 | 0.014 | 0.000 | 0.000 | 0.000 | 0.000 | 0.000 | 0.000 | 0.000 |
| **g__Kordiimonas** | 0.000 | 0.000 | 0.000 | 0.000 | 0.014 | 0.000 | 0.000 | 0.000 | 0.000 | 0.000 | 0.000 | 0.000 |
| **g__Vogesella** | 0.000 | 0.000 | 0.000 | 0.000 | 0.014 | 0.000 | 0.000 | 0.000 | 0.000 | 0.000 | 0.000 | 0.000 |
| **g__Aliagarivorans** | 0.000 | 0.000 | 0.000 | 0.000 | 0.014 | 0.000 | 0.000 | 0.000 | 0.000 | 0.000 | 0.000 | 0.000 |
| **c__Opitutae** | 0.000 | 0.000 | 0.000 | 0.000 | 0.014 | 0.000 | 0.000 | 0.000 | 0.000 | 0.000 | 0.000 | 0.000 |
| **g__Coraliomargarita** | 0.000 | 0.000 | 0.000 | 0.000 | 0.014 | 0.000 | 0.000 | 0.000 | 0.000 | 0.000 | 0.000 | 0.000 |
| **g__Amaricoccus** | 0.000 | 0.010 | 0.000 | 0.000 | 0.000 | 0.000 | 0.000 | 0.000 | 0.000 | 0.000 | 0.000 | 0.003 |
| **g__Staphylococcus** | 0.000 | 0.000 | 0.000 | 0.000 | 0.000 | 0.000 | 0.004 | 0.004 | 0.005 | 0.000 | 0.000 | 0.000 |
| **f__Victivallaceae** | 0.000 | 0.000 | 0.005 | 0.000 | 0.000 | 0.000 | 0.000 | 0.000 | 0.000 | 0.007 | 0.000 | 0.000 |
| **g__Gramella** | 0.000 | 0.000 | 0.000 | 0.012 | 0.000 | 0.000 | 0.000 | 0.000 | 0.000 | 0.000 | 0.000 | 0.000 |
| **g__BD2-13** | 0.000 | 0.000 | 0.000 | 0.012 | 0.000 | 0.000 | 0.000 | 0.000 | 0.000 | 0.000 | 0.000 | 0.000 |
| **f__C111** | 0.000 | 0.000 | 0.011 | 0.000 | 0.000 | 0.000 | 0.000 | 0.000 | 0.000 | 0.000 | 0.000 | 0.000 |
| **f__Nostocaceae** | 0.000 | 0.000 | 0.011 | 0.000 | 0.000 | 0.000 | 0.000 | 0.000 | 0.000 | 0.000 | 0.000 | 0.000 |
| **g__Leuconostoc** | 0.000 | 0.000 | 0.011 | 0.000 | 0.000 | 0.000 | 0.000 | 0.000 | 0.000 | 0.000 | 0.000 | 0.000 |
| **g__Salinimicrobium** | 0.000 | 0.000 | 0.000 | 0.000 | 0.000 | 0.000 | 0.000 | 0.011 | 0.000 | 0.000 | 0.000 | 0.000 |
| **g__Bradyrhizobium** | 0.000 | 0.000 | 0.000 | 0.000 | 0.000 | 0.000 | 0.000 | 0.011 | 0.000 | 0.000 | 0.000 | 0.000 |
| **g__Ensifer** | 0.000 | 0.000 | 0.000 | 0.000 | 0.000 | 0.000 | 0.000 | 0.011 | 0.000 | 0.000 | 0.000 | 0.000 |
| **g__Granulicatella** | 0.000 | 0.000 | 0.000 | 0.000 | 0.000 | 0.011 | 0.000 | 0.000 | 0.000 | 0.000 | 0.000 | 0.000 |
| **c__RB25** | 0.000 | 0.010 | 0.000 | 0.000 | 0.000 | 0.000 | 0.000 | 0.000 | 0.000 | 0.000 | 0.000 | 0.000 |
| **f__Nocardioidaceae** | 0.000 | 0.010 | 0.000 | 0.000 | 0.000 | 0.000 | 0.000 | 0.000 | 0.000 | 0.000 | 0.000 | 0.000 |
| **g__KSA1** | 0.000 | 0.010 | 0.000 | 0.000 | 0.000 | 0.000 | 0.000 | 0.000 | 0.000 | 0.000 | 0.000 | 0.000 |
| **g__Prochlorococcus** | 0.000 | 0.010 | 0.000 | 0.000 | 0.000 | 0.000 | 0.000 | 0.000 | 0.000 | 0.000 | 0.000 | 0.000 |
| **g__Brevibacillus** | 0.000 | 0.010 | 0.000 | 0.000 | 0.000 | 0.000 | 0.000 | 0.000 | 0.000 | 0.000 | 0.000 | 0.000 |
| **g__Rummeliibacillus** | 0.000 | 0.010 | 0.000 | 0.000 | 0.000 | 0.000 | 0.000 | 0.000 | 0.000 | 0.000 | 0.000 | 0.000 |
| **g__Pseudobutyrivibrio** | 0.000 | 0.010 | 0.000 | 0.000 | 0.000 | 0.000 | 0.000 | 0.000 | 0.000 | 0.000 | 0.000 | 0.000 |
| **g__Filifactor** | 0.000 | 0.010 | 0.000 | 0.000 | 0.000 | 0.000 | 0.000 | 0.000 | 0.000 | 0.000 | 0.000 | 0.000 |
| **g__Subdoligranulum** | 0.000 | 0.010 | 0.000 | 0.000 | 0.000 | 0.000 | 0.000 | 0.000 | 0.000 | 0.000 | 0.000 | 0.000 |
| **g__Balneimonas** | 0.000 | 0.010 | 0.000 | 0.000 | 0.000 | 0.000 | 0.000 | 0.000 | 0.000 | 0.000 | 0.000 | 0.000 |
| **g__Chelativorans** | 0.000 | 0.010 | 0.000 | 0.000 | 0.000 | 0.000 | 0.000 | 0.000 | 0.000 | 0.000 | 0.000 | 0.000 |
| **f__NB1-i** | 0.000 | 0.010 | 0.000 | 0.000 | 0.000 | 0.000 | 0.000 | 0.000 | 0.000 | 0.000 | 0.000 | 0.000 |
| **g__HTCC2207** | 0.000 | 0.010 | 0.000 | 0.000 | 0.000 | 0.000 | 0.000 | 0.000 | 0.000 | 0.000 | 0.000 | 0.000 |
| **g__Azotobacter** | 0.000 | 0.010 | 0.000 | 0.000 | 0.000 | 0.000 | 0.000 | 0.000 | 0.000 | 0.000 | 0.000 | 0.000 |
| **g__Turneriella** | 0.000 | 0.010 | 0.000 | 0.000 | 0.000 | 0.000 | 0.000 | 0.000 | 0.000 | 0.000 | 0.000 | 0.000 |
| **g__Aurantimonas** | 0.000 | 0.000 | 0.000 | 0.000 | 0.000 | 0.000 | 0.000 | 0.000 | 0.000 | 0.000 | 0.000 | 0.010 |
| **f__Methylocystaceae** | 0.000 | 0.000 | 0.000 | 0.000 | 0.000 | 0.000 | 0.000 | 0.000 | 0.000 | 0.000 | 0.000 | 0.010 |
| **g__Geodermatophilus** | 0.010 | 0.000 | 0.000 | 0.000 | 0.000 | 0.000 | 0.000 | 0.000 | 0.000 | 0.000 | 0.000 | 0.000 |
| **o__GCA004** | 0.010 | 0.000 | 0.000 | 0.000 | 0.000 | 0.000 | 0.000 | 0.000 | 0.000 | 0.000 | 0.000 | 0.000 |
| **g__Chloronema** | 0.010 | 0.000 | 0.000 | 0.000 | 0.000 | 0.000 | 0.000 | 0.000 | 0.000 | 0.000 | 0.000 | 0.000 |
| **c__Ktedonobacteria** | 0.010 | 0.000 | 0.000 | 0.000 | 0.000 | 0.000 | 0.000 | 0.000 | 0.000 | 0.000 | 0.000 | 0.000 |
| **g__Geobacillus** | 0.010 | 0.000 | 0.000 | 0.000 | 0.000 | 0.000 | 0.000 | 0.000 | 0.000 | 0.000 | 0.000 | 0.000 |
| **g__Geomicrobium** | 0.010 | 0.000 | 0.000 | 0.000 | 0.000 | 0.000 | 0.000 | 0.000 | 0.000 | 0.000 | 0.000 | 0.000 |
| **g__Alloiococcus** | 0.010 | 0.000 | 0.000 | 0.000 | 0.000 | 0.000 | 0.000 | 0.000 | 0.000 | 0.000 | 0.000 | 0.000 |
| **g__Anaerostipes** | 0.010 | 0.000 | 0.000 | 0.000 | 0.000 | 0.000 | 0.000 | 0.000 | 0.000 | 0.000 | 0.000 | 0.000 |
| **o__Fusobacteriales** | 0.000 | 0.000 | 0.000 | 0.000 | 0.000 | 0.005 | 0.004 | 0.000 | 0.000 | 0.000 | 0.000 | 0.000 |
| **c__BD4-9** | 0.010 | 0.000 | 0.000 | 0.000 | 0.000 | 0.000 | 0.000 | 0.000 | 0.000 | 0.000 | 0.000 | 0.000 |
| **g__Rhodobacter** | 0.010 | 0.000 | 0.000 | 0.000 | 0.000 | 0.000 | 0.000 | 0.000 | 0.000 | 0.000 | 0.000 | 0.000 |
| **f__HTCC2089** | 0.010 | 0.000 | 0.000 | 0.000 | 0.000 | 0.000 | 0.000 | 0.000 | 0.000 | 0.000 | 0.000 | 0.000 |
| **g__Pyramidobacter** | 0.010 | 0.000 | 0.000 | 0.000 | 0.000 | 0.000 | 0.000 | 0.000 | 0.000 | 0.000 | 0.000 | 0.000 |
| **o__HA64** | 0.010 | 0.000 | 0.000 | 0.000 | 0.000 | 0.000 | 0.000 | 0.000 | 0.000 | 0.000 | 0.000 | 0.000 |
| **g__Ornithinimicrobium** | 0.000 | 0.000 | 0.000 | 0.000 | 0.000 | 0.000 | 0.000 | 0.000 | 0.010 | 0.000 | 0.000 | 0.000 |
| **g__Serinicoccus** | 0.000 | 0.000 | 0.000 | 0.000 | 0.000 | 0.000 | 0.000 | 0.000 | 0.010 | 0.000 | 0.000 | 0.000 |
| **g__Lactococcus** | 0.000 | 0.000 | 0.000 | 0.000 | 0.000 | 0.000 | 0.000 | 0.000 | 0.010 | 0.000 | 0.000 | 0.000 |
| **g__Marihabitans** | 0.000 | 0.000 | 0.000 | 0.000 | 0.000 | 0.000 | 0.009 | 0.000 | 0.000 | 0.000 | 0.000 | 0.000 |
| **f__Actinosynnemataceae** | 0.000 | 0.000 | 0.000 | 0.000 | 0.000 | 0.000 | 0.000 | 0.000 | 0.000 | 0.000 | 0.008 | 0.000 |
| **g__Wandonia** | 0.000 | 0.000 | 0.000 | 0.000 | 0.000 | 0.000 | 0.000 | 0.000 | 0.000 | 0.000 | 0.008 | 0.000 |
| **g__Kordia** | 0.000 | 0.000 | 0.000 | 0.000 | 0.000 | 0.000 | 0.000 | 0.000 | 0.000 | 0.000 | 0.008 | 0.000 |
| **g__Olleya** | 0.000 | 0.000 | 0.000 | 0.000 | 0.000 | 0.000 | 0.000 | 0.000 | 0.000 | 0.000 | 0.008 | 0.000 |
| **g__Winogradskyella** | 0.000 | 0.000 | 0.000 | 0.000 | 0.000 | 0.000 | 0.000 | 0.000 | 0.000 | 0.000 | 0.008 | 0.000 |
| **o__DRC31** | 0.000 | 0.000 | 0.000 | 0.000 | 0.000 | 0.000 | 0.000 | 0.000 | 0.000 | 0.000 | 0.008 | 0.000 |
| **c__4C0d-2** | 0.000 | 0.000 | 0.000 | 0.000 | 0.000 | 0.000 | 0.000 | 0.000 | 0.000 | 0.000 | 0.008 | 0.000 |
| **g__Phascolarctobacterium** | 0.000 | 0.000 | 0.000 | 0.000 | 0.000 | 0.000 | 0.000 | 0.000 | 0.000 | 0.000 | 0.008 | 0.000 |
| **o__Aeromonadales** | 0.000 | 0.000 | 0.000 | 0.000 | 0.000 | 0.000 | 0.000 | 0.000 | 0.000 | 0.000 | 0.008 | 0.000 |
| **g__Oleiphilus** | 0.000 | 0.000 | 0.000 | 0.000 | 0.000 | 0.000 | 0.000 | 0.000 | 0.000 | 0.000 | 0.008 | 0.000 |
| **p__Verrucomicrobia** | 0.000 | 0.000 | 0.000 | 0.000 | 0.000 | 0.000 | 0.000 | 0.000 | 0.000 | 0.000 | 0.008 | 0.000 |
| **o__Cenarchaeales** | 0.000 | 0.000 | 0.000 | 0.000 | 0.000 | 0.000 | 0.000 | 0.007 | 0.000 | 0.000 | 0.000 | 0.000 |
| **o__CV90** | 0.000 | 0.000 | 0.000 | 0.000 | 0.000 | 0.000 | 0.000 | 0.007 | 0.000 | 0.000 | 0.000 | 0.000 |
| **g__Flavisolibacter** | 0.000 | 0.000 | 0.000 | 0.000 | 0.000 | 0.000 | 0.000 | 0.000 | 0.000 | 0.007 | 0.000 | 0.000 |
| **o__[Roseiflexales]** | 0.000 | 0.000 | 0.000 | 0.000 | 0.000 | 0.000 | 0.000 | 0.000 | 0.000 | 0.007 | 0.000 | 0.000 |
| **g__[Eubacterium]** | 0.000 | 0.000 | 0.000 | 0.000 | 0.000 | 0.000 | 0.000 | 0.000 | 0.000 | 0.007 | 0.000 | 0.000 |
| **g__Planctomyces** | 0.000 | 0.000 | 0.000 | 0.000 | 0.000 | 0.000 | 0.000 | 0.000 | 0.000 | 0.007 | 0.000 | 0.000 |
| **o__Kiloniellales** | 0.000 | 0.000 | 0.000 | 0.000 | 0.000 | 0.000 | 0.000 | 0.000 | 0.000 | 0.007 | 0.000 | 0.000 |
| **g__Porphyrobacter** | 0.000 | 0.000 | 0.000 | 0.000 | 0.000 | 0.000 | 0.000 | 0.000 | 0.000 | 0.007 | 0.000 | 0.000 |
| **g__Kaistobacter** | 0.000 | 0.000 | 0.000 | 0.000 | 0.000 | 0.000 | 0.000 | 0.000 | 0.000 | 0.007 | 0.000 | 0.000 |
| **f__Cystobacteraceae** | 0.000 | 0.000 | 0.000 | 0.000 | 0.000 | 0.000 | 0.000 | 0.000 | 0.000 | 0.007 | 0.000 | 0.000 |
| **g__Pseudidiomarina** | 0.000 | 0.000 | 0.000 | 0.000 | 0.000 | 0.000 | 0.000 | 0.000 | 0.000 | 0.007 | 0.000 | 0.000 |
| **f__Cardiobacteriaceae** | 0.000 | 0.000 | 0.000 | 0.000 | 0.000 | 0.000 | 0.000 | 0.000 | 0.000 | 0.007 | 0.000 | 0.000 |
| **f__Aurantimonadaceae** | 0.000 | 0.000 | 0.000 | 0.000 | 0.000 | 0.000 | 0.000 | 0.000 | 0.000 | 0.000 | 0.000 | 0.007 |
| **g__Tetrathiobacter** | 0.000 | 0.000 | 0.000 | 0.000 | 0.000 | 0.000 | 0.000 | 0.000 | 0.000 | 0.000 | 0.000 | 0.007 |
| **f__ACK-M1** | 0.000 | 0.000 | 0.005 | 0.000 | 0.000 | 0.000 | 0.000 | 0.000 | 0.000 | 0.000 | 0.000 | 0.000 |
| **f__p-2534-18B5** | 0.000 | 0.000 | 0.005 | 0.000 | 0.000 | 0.000 | 0.000 | 0.000 | 0.000 | 0.000 | 0.000 | 0.000 |
| **g__Croceitalea** | 0.000 | 0.000 | 0.005 | 0.000 | 0.000 | 0.000 | 0.000 | 0.000 | 0.000 | 0.000 | 0.000 | 0.000 |
| **f__Christensenellaceae** | 0.000 | 0.000 | 0.005 | 0.000 | 0.000 | 0.000 | 0.000 | 0.000 | 0.000 | 0.000 | 0.000 | 0.000 |
| **f__Kordiimonadaceae** | 0.000 | 0.000 | 0.005 | 0.000 | 0.000 | 0.000 | 0.000 | 0.000 | 0.000 | 0.000 | 0.000 | 0.000 |
| **f__Pelagibacteraceae** | 0.000 | 0.000 | 0.005 | 0.000 | 0.000 | 0.000 | 0.000 | 0.000 | 0.000 | 0.000 | 0.000 | 0.000 |
| **g__Pelagibacter** | 0.000 | 0.000 | 0.005 | 0.000 | 0.000 | 0.000 | 0.000 | 0.000 | 0.000 | 0.000 | 0.000 | 0.000 |
| **g__Denitromonas** | 0.000 | 0.000 | 0.005 | 0.000 | 0.000 | 0.000 | 0.000 | 0.000 | 0.000 | 0.000 | 0.000 | 0.000 |
| **g__ZA3312c** | 0.000 | 0.000 | 0.005 | 0.000 | 0.000 | 0.000 | 0.000 | 0.000 | 0.000 | 0.000 | 0.000 | 0.000 |
| **g__Kineosphaera** | 0.000 | 0.000 | 0.000 | 0.000 | 0.000 | 0.005 | 0.000 | 0.000 | 0.000 | 0.000 | 0.000 | 0.000 |
| **g__Abiotrophia** | 0.000 | 0.000 | 0.000 | 0.000 | 0.000 | 0.005 | 0.000 | 0.000 | 0.000 | 0.000 | 0.000 | 0.000 |
| **f__Carnobacteriaceae** | 0.000 | 0.000 | 0.000 | 0.000 | 0.000 | 0.005 | 0.000 | 0.000 | 0.000 | 0.000 | 0.000 | 0.000 |
| **g__Thiothrix** | 0.000 | 0.000 | 0.000 | 0.000 | 0.000 | 0.005 | 0.000 | 0.000 | 0.000 | 0.000 | 0.000 | 0.000 |
| **o__iii1-15** | 0.000 | 0.000 | 0.000 | 0.000 | 0.000 | 0.000 | 0.000 | 0.000 | 0.005 | 0.000 | 0.000 | 0.000 |
| **f__[Amoebophilaceae]** | 0.000 | 0.000 | 0.000 | 0.000 | 0.000 | 0.000 | 0.000 | 0.000 | 0.005 | 0.000 | 0.000 | 0.000 |
| **g__Synechococcus** | 0.000 | 0.000 | 0.000 | 0.000 | 0.000 | 0.000 | 0.000 | 0.000 | 0.005 | 0.000 | 0.000 | 0.000 |
| **g__Marinococcus** | 0.000 | 0.000 | 0.000 | 0.000 | 0.000 | 0.000 | 0.000 | 0.000 | 0.005 | 0.000 | 0.000 | 0.000 |
| **g__Nitrospira** | 0.000 | 0.000 | 0.000 | 0.000 | 0.000 | 0.000 | 0.000 | 0.000 | 0.005 | 0.000 | 0.000 | 0.000 |
| **f__Thermaceae** | 0.000 | 0.000 | 0.000 | 0.000 | 0.000 | 0.000 | 0.000 | 0.000 | 0.005 | 0.000 | 0.000 | 0.000 |
| **p__Spirochaetes** | 0.000 | 0.000 | 0.000 | 0.000 | 0.000 | 0.000 | 0.004 | 0.000 | 0.000 | 0.000 | 0.000 | 0.000 |
| **f__Bifidobacteriaceae** | 0.000 | 0.000 | 0.000 | 0.000 | 0.000 | 0.000 | 0.000 | 0.004 | 0.000 | 0.000 | 0.000 | 0.000 |
| **f__Gaiellaceae** | 0.000 | 0.000 | 0.000 | 0.000 | 0.000 | 0.000 | 0.000 | 0.004 | 0.000 | 0.000 | 0.000 | 0.000 |
| **g__Alkaliflexus** | 0.000 | 0.000 | 0.000 | 0.000 | 0.000 | 0.000 | 0.000 | 0.004 | 0.000 | 0.000 | 0.000 | 0.000 |
| **p__FBP** | 0.000 | 0.000 | 0.000 | 0.000 | 0.000 | 0.000 | 0.000 | 0.004 | 0.000 | 0.000 | 0.000 | 0.000 |
| **g__SMB53** | 0.000 | 0.000 | 0.000 | 0.000 | 0.000 | 0.000 | 0.000 | 0.004 | 0.000 | 0.000 | 0.000 | 0.000 |
| **g__Sinorhizobium** | 0.000 | 0.000 | 0.000 | 0.000 | 0.000 | 0.000 | 0.000 | 0.004 | 0.000 | 0.000 | 0.000 | 0.000 |
| **g__Diaphorobacter** | 0.000 | 0.000 | 0.000 | 0.000 | 0.000 | 0.000 | 0.000 | 0.004 | 0.000 | 0.000 | 0.000 | 0.000 |
| **g__Halorhodospira** | 0.000 | 0.000 | 0.000 | 0.000 | 0.000 | 0.000 | 0.000 | 0.004 | 0.000 | 0.000 | 0.000 | 0.000 |
| **o__Methylococcales** | 0.000 | 0.000 | 0.000 | 0.000 | 0.000 | 0.000 | 0.000 | 0.004 | 0.000 | 0.000 | 0.000 | 0.000 |
| **c__Deinococci** | 0.000 | 0.000 | 0.000 | 0.000 | 0.000 | 0.000 | 0.000 | 0.004 | 0.000 | 0.000 | 0.000 | 0.000 |
| **g__Roseivirga** | 0.000 | 0.000 | 0.000 | 0.000 | 0.000 | 0.000 | 0.000 | 0.000 | 0.000 | 0.000 | 0.000 | 0.003 |
| **g__Pedobacter** | 0.000 | 0.000 | 0.000 | 0.000 | 0.000 | 0.000 | 0.000 | 0.000 | 0.000 | 0.000 | 0.000 | 0.003 |
| **g__Desulfococcus** | 0.000 | 0.000 | 0.000 | 0.000 | 0.000 | 0.000 | 0.000 | 0.000 | 0.000 | 0.000 | 0.000 | 0.003 |
